# Sex-Differences in Aortic Stenosis: Mechanistic Insights and Clinical Implications

**DOI:** 10.3389/fcvm.2022.818371

**Published:** 2022-02-24

**Authors:** Lara Matilla, Mattie Garaikoetxea, Vanessa Arrieta, Amaia García-Peña, Amaya Fernández-Celis, Adela Navarro, Alicia Gainza, Virginia Álvarez, Rafael Sádaba, Eva Jover, Natalia López-Andrés

**Affiliations:** Cardiovascular Translational Research, Navarrabiomed, Complejo Hospitalario de Navarra (CHN), Universidad Pública de Navarra (UPNA), IdiSNA, Pamplona, Spain

**Keywords:** aortic stenosis, sex, valve interstitial cells, extracellular matrix, remodeling

## Abstract

**Objective:**

We aim to analyse sex-specific differences in aortic valves (AVs) and valve interstitial cells (VICs) from aortic stenosis (AS) patients.

**Approach and Results:**

238 patients with severe AS undergoing surgical valve replacement were recruited. Two hundred and two AVs (39.1% women) were used for *ex vivo* analyses and 36 AVs (33.3% women) for *in vitro* experiments. AVs from men presented increased levels of the inflammatory molecules interleukin (IL)-1β, IL-6, Rantes, and CD45. Oxidative stress (eNOS, myeloperoxidase, malondialdehyde and nitrotyrosine) was upregulated in male AVs. Concerning fibrosis, similar levels of collagen type I, decreased levels of collagen type III and enhanced fibronectin, active Lox-1 and syndecan-1 expressions were found in AVs from men compared with women. Extracellular matrix (ECM) remodeling was characterized by reduced metalloproteinase-1 and 9 expression and increased tissue inhibitor of metalloproteinase-2 expression in male AVs. Importantly, osteogenic markers (bone morphogenetic protein-9, Rank-L, osteopontin, periostin, osteocalcin and Sox-9) and apoptosis (Bax, Caspase 3, p53, and PARP1) were enhanced in AVs from men as compared to women. Isolated male VICs presented higher myofibroblast-like phenotype than female VICs. Male VICs exhibited increased inflammatory, oxidative stress, fibrotic, apoptosis and osteogenic differentiation markers.

**Conclusions:**

Our results suggest that the mechanisms driving the pathogenesis of AS could be different in men and women. Male AVs and isolated VICs presented more inflammation, oxidative stress, ECM remodeling and calcification as compared to those from women. A better knowledge of the pathophysiological pathways in AVs and VICs will allow the development of sex-specific options for the treatment of AS.

## Highlights

- Our study characterizes for the first time the sex-related histological and molecular expression patterns of inflammatory, oxidative stress, fibrosis, extracellular matrix deposition, calcification, and apoptosis markers in aortic valves.
- Male aortic valves and isolated valve interstitial cells presented more inflammation, oxidative stress, extracellular matrix deposition and calcification as compared to those from women.
- Aortic valves and isolated valve interstitial cells from women exhibited more collagen type III content accompanied by increased extracellular matrix degradation markers.

## Introduction

Aortic stenosis (AS) is the most prevalent form of heart valve disease. It is present in 2% to 7% of individuals older than 65 years (12.4% in European population >75) and the 2-year mortality rate rises up to 50% when AS becomes symptomatic (1, 2). In 2017, about 12.6 million cases of AS were reported, with an estimated mortality rate of 0.8% (3).

AS is a degenerative and progressive disease, leading to aortic valve (AV) dysfunction and poor clinical outcomes. Valve leaflet fibrosis and calcification are the two main hallmark lesions of AS. It is accepted that AS pathogenesis is a biologically active process sharing several commonalities with atherosclerosis including complex proliferative and inflammatory changes (4). Valve resident cells, including the valve interstitial and endothelial cells (VIC and VEC, respectively) play a key role in the pathogenesis of AS (5). VICs are a heterogeneous cell population which under pathological conditions become myofibroblasts-like cells. As a result, misbalanced extracellular matrix (ECM) synthesis and degradation, namely ECM remodeling, and inflammation trigger VICs apoptosis, oxidative stress, all of them leading to extensive calcification of the AV cusps (5, 6). Despite recent advances in the knowledge of the pathophysiology of AS, there are no current pharmacologic-based therapies available, only the replacement of the naïve valve (7).

Sex is an important modulator of pathological processes implicated in the development of AS, although it remains largely unexplored (8). Recently, sexual dimorphism has been described in AVs from patients with AS. AVs from women presented more fibrosis and dense connective tissue with less calcification than men for the same degree of AS severity (9–11). In isolated porcine VICs, a different pattern of mRNA expression has been defined between sexes suggesting that different pathological mechanisms may contribute to the sex-related discrepancies (6). However, it remains unclear how cellular and molecular mechanisms orchestrate AS pathophysiology in men and women. The aim of our study was to analyse more in depth the sexual dimorphism in patients with AS, focusing on histological, molecular and cellular alterations.

## Materials and Methods

### Clinical Cohort

This prospective and observational study included a total of 238 patients with severe AS referred to Complejo Hospitalario de Navarra for surgical AV replacement from June 2013 to October 2020. These 238 patients were divided into two cohorts dedicated to (i) histological and molecular analyses (*n* = 202) and (ii) isolation of primary culture VICs for *in vitro* experiments (*n* = 36). AS was defined following current guidelines (12) as aortic valve area ≤ 1 cm^2^ and/or transaortic mean pressure gradient >40 mm Hg. Exclusion criteria were: moderate or severe concomitant valvular disease, malignant tumor, infective endocarditis, diabetes mellitus and chronic inflammatory diseases.

All patients were evaluated by transthoracic echocardiography. Venous blood was drawn for measurement of brain natriuretic peptide (BNP) and other routine laboratory parameters on admission for surgery.

Informed consent was obtained from each patient. The study protocol conforms to the ethical guidelines of the 1975 Declaration of Helsinki as reflected in a previous approval by the institution's human research committee (Comité Ético de Experimentación Clínica. Gobierno de Navarra, Departamento de Salud; Ethics numbers 17/2013 and PI2019/59).

### *Ex vivo* Experiments

We collected a total of 202 valves (79 from women and 123 from men). AVs harvested from AS patients (*n* = 202, 39.11% women) undergoing elective surgical valve replacement were dissected into two pieces. One part was dedicated to protein and RNA extractions and another one was paraffin-embedded for histological and immunohistochemistry analyses. Moreover, whole AVs (*n* = 36, 33.33% women) were dedicated to VIC isolation in this manuscript. All the experiments were performed by 2 different blinded observers.

### Cell Isolation and Culture

Human VICs were isolated from 36 AVs (12 women and 24 men) obtained during surgical AV replacement. VICs from each patient were isolated and individually assayed, as previously described (13). In brief, AVs were minced and enzymatically digested into 2 mL of buffered-collagenase type 2 (240 U/mg of tissue) for 1 h, and were pelleted by centrifugation. VICs were cultured in DMEM F-12 medium (Gibco) supplemented with 20% fetal bovine serum (FBS) (Gibco), 1% Penicilin/Streptomicin (Lonza), 5 μg/ml insulin (Sigma Aldrich) and 10 ng/ml of fibroblast growth factor (FGF-2) (Novus Biological) at 37°C and 5% CO_2_ in a saturation humidified incubator (Panasonic). The VIC phenotype of isolated cells was confirmed at passage 1 by vimentin and alpha-smooth muscle actin (α-SMA) immunocytochemistry (Supplementary Figures 1A,B). Experiments were performed in serum-starvation conditions (1% FBS) in multiwell plates (Sarstedt). All experiments were carried out in VICs at passage 3–4. At least, four biological replicates (donors) *per* sex were used in each experiment and 3–8 technical replicates were performed to guarantee the availability of sample to be lately assayed using different techniques. The number of biological replicates (donors) and technical replicates is specified in the Figure Legends.

### Histology and Immunohistochemistry Evaluation

Histological determinations in whole AVs were performed in 5 μm-thick paraffin-embedded serial sections following the protocol of Leica BOND-Polymer Re-fine Detection automatic immunostainer (Leica). All solutions were filled into the bottle-Bond Open Container (Leica) and registered on computer using the Leica Biosystem program. The immunostaining program protocol include: Fixative solution, Bond wash solution, Blocking with common immunohistochemistry blocker and incubated with the primary antibody for Rantes (Santa Cruz Biotechnology), CD45 (Santa Cruz Biotechnology), CD68 (Santa Cruz Biotechnology), endothelial nitric oxide synthase [eNOS (BD biosciences)], carboxymethyl Lysine (Abcam), malondialdehyde (Abcam), Nitrotyrosine (Santa Cruz Biotechnology), matrix metalloproteinases [MMP-1 (Abcam); MMP-2 (Santa Cruz Biotechnology); MMP-9 (Santa Cruz Biotechnology)], tissue inhibitor of metalloproteinases [TIMP-2 (Santa Cruz Biotechnology)], periostin (Santa Cruz Biotechnology), osteocalcin (Santa Cruz Biotechnology), Bcl-2-associated X [Bax (Santa Cruz Biotechnology)], caspase 3 (Cell Signaling), p53 (Santa Cruz Biotechnology) and amyloid-β(Dako). After primary antibody incubation, slides were incubated with secondary poly-HRP-IgG. The signal was revealed by using DAB substrate. Incubation with no primary antibody was carried out in negative controls.

All tissues were hydrated in water. For proteoglycan and collagen identification, slides were incubated with alcian-blue (Sigma-Aldrich) for 20 min and with 1% Sirius red in picric acid for 30 min thereafter. Elastic stain kit was used to image elastic fibers according to the manufacturer's instructions (Sigma-Aldrich). Inflammatory infiltrates were visualized by haematoxylin/eosin staining (Panreac/Bio-optica). Movat pentachrome staining, used to assess general histoanatomical features of the AVs, was performed following the manufacturer's instructions (Abcam). Calcification was analyzed by alizarin red staining (2% in aqueous solution, pH: 4.1–4.3 with NH_4_OH, Sigma-Aldrich). Congo red staining was performed following the manufacturer's instructions (Merck) to evidence β-amyloid fiber deposition.

Histological and immunohistochemistry preparations were imaged using bright field or polarized light in an automated image analysis system, as appropriate (Nikon). Digital image analyses were additionally performed for a further histoanatomical characterization. In brief, arbitrary fields per section were imaged at 50 or 400X magnification, as appropriate. The content of molecular and histological targets (e.g., thin and thick collagen fibers, calcium deposits or mature bone) were quantified using Image J software by performing a binary conversion of the images on red channel to quantify the positive % of area occupied by these targets. All quantifications were normalized to the area of the tissue analyzed. All the experiments were performed by 2 different blinded observers.

In the figures the most representative image were shown.

### ELISA

Interleukins (IL-6; IL-1β), C-C motif chemokine ligand 2 (CCL2), Rantes, collagen-1, fibronectin, decorin, lumican, syndecan-1, aggrecan, bone morphogenetic proteins (BMP-2, BMP-4, BMP-9), receptor activator of NF-kappaB ligand (Rank-L), osteopontin, periostin, osteocalcin, metalloproteinases (MMP-1, MMP-2, MMP-9), tissue inhibitors of MMPs (TIMP-1, TIMP-2), myeloperoxidase, CD14, eNOS and osteoprotegerin were measured in AVs extracts and cells supernatants according to the manufacturer's instructions (R&D Systems). Caspase 3 activity was further analyzed in VIC protein lysates following the manufacturer's instructions (R&D Systems). For explanted AV, equal micrograms of total protein (tissue lysates) were loaded and assayed by ELISA; for *in vitro* samples, equal volumes of cell supernatants were used and were thereafter normalized by the total micrograms of protein collected from the respective cell monolayers. Results from ELISA analyses have been plotted as fold-change with women as calibrator or reference group. Alongside the manuscript both the net concentration and the fold-change means are discussed for further information.

### Western Blot Analysis (WB)

Aliquots of 20 μg of total proteins were prepared and electrophoresed from AV and VICs extracts on SDS polyacrylamide gels (4–15% polyacrylamide, Mini-PROTEANTGX Stain-Free, BioRad) and transferred to Hybond-C Extra nitrocellulose membranes (BioRad). Membranes were incubated with primary antibodies for: lysil oxydase (Lox-1), SRY (sex-determining region Y)-box 9 (Sox-9), malondialdehyde, Bax, Caspase-3, p53, Poly [ADP-ribose] polymerase 1 (PARP1), CD45, CD68, CD80, carboxymethyl-lysine, Nitrotyrosine, superoxide dismutase-1, peroxiredoxin IV, Catalase, runt-related transcription factor 2 (Runx2), lumican, fibronectin, transforming growth factor- β (TGF-β, collagen type III and β-actin; and with secondary antibodies for mouse and rabbit (GE Healthcare). Blot densitometry analyses were performed using Image Lab software. β-actin and stain free were used as loading controls for normalization and the net band densitometry was expressed as arbitrary units (AU). Positive blots were detected with a chemiluminiscence method (ECL, Amersham Biosciences) and images acquired with Chemidoc MP Imaging system (Bio-Rad). All western blots were performed at least in triplicate for each experimental condition. Semiquantitative analyses were performed by band densitometry using Image Lab software (Bio-Rad). Alongside the manuscript both the net AU and the fold-change means are discussed for further information.

### Real-Time Reverse Transcription PCR

Total RNA from cells and AVs was extracted with Trizol Reagent (Canvas). First strand cDNA was synthesized according to the manufacturer's instructions (Bio-Rad). Quantitative PCR analysis was then performed with SYBR green PCR technology (Bio-Rad) (Supplementary Major Resource Table). Relative quantification was achieved with MyiQ (Bio-Rad) software according to the manufacturer's instructions. Data were normalized to18S, HPRT, β-actin and GADPH levels, and expressed as fold-change relative to women. All PCRs were performed at least in triplicate for each experimental condition. Results have been plotted as fold-change with women as calibrator or reference group.

### Zymography

Aliquots of 20 μg of overall to proteins were settled on a 10% SDS polyacrylamide gel containing 0.3% gelatin. The gel was washed three times for 15 min with a solution of 2.5% Triton X 100 to remove SDS and renature the proteins. Then we incubate overnight at 37°C in 1 mol/l Tris-HCl, pH 7.5 with 1 mol/l CaCl_2_ and 5 mol/l NaCl to promote gelatinase activity. Gels were fixed in 40% methanol and 10% acetic acid and stained for 30 min in 0.25% Coomassie blue R-250 to analyse proteolytic activity of MMPs (14). Semiquantitative analyses were performed by band densitometry using Image Lab software (Bio-Rad). Fold changes in band densitometries have been expressed in arbitrary units (AU). Results have been plotted as fold-change with women as calibrator or reference group. The net densitometry and the fold-change means are discussed alongside the manuscript for further information.

### Statistical Analyses

The characteristics of the patients were summarized using frequencies and percentages, means and standard deviations (SD). Data normality was assessed through Shapiro-Wilk's test and Kolmogorov-Smirnov (with Lilliefors *p*-value). Quantitative variables were analyzed by T student test or Mann-Whitney U test if the normality was not met. The effects sex on demographic, clinical and analytic related variables were assessed in two steps. First, univariate lineal regressions models were fitted for all continuous variables. Similarly univariate logistic regression models were used to estimate the odds ratios of categorical variables. These steps allowed to identify the variables that differed significantly between sexes and that therefore could be potential confounders. In order to adjust for the potential confounders found by the univariate analyses, a second analysis step was added where the adjusted effect of sex over the analyzed variables was modeled using lineal and logistic multivariate regression models, as appropriate. Briefly, based on the magnitude of the effect and the *p*-values calculated in the univariate models, age, statin use and total cholesterol levels were used as covariates of the multivariate models. For each variable, an adjusted model was fitted to calculate odds ratio (OR). The absence of multicollinearity was guaranteed making use of the Variance Inflation Factor for each independent variable. The adjusted *p*-value was therefore calculated as de *p*-value of the sex covariate in the multivariate model that was adjusted for the variables chosen in step one. A *p*-value of <0.05 was considered statistically significant. All analyses in the clinical cohort were performed using the R statistical package, v. 3.6 (R Foundation for Statistical Computing. Vienna, Austria). GraphPad Software Inc. was used for *in vitro* analyses.

## Results

### Clinical Parameters in AS Patients

Two different cohorts of AS patients were included in this study. Cohort 1 was dedicated to perform histological and molecular analyses in the AVs. Baseline clinical and demographical characteristics of cohort 1 are summarized in Table 1. A total amount of 202 patients (39.1% women) were recruited. Women enrolled in this study were significantly older than men (Table 1). Moreover, lower height, weight and body surface were reported in women as compared to men. Among the 202 patients, 45.7% had bicuspid aortic valve (BAV) ([46.2%] women, [45.5%] men). Lipid profiling revealed higher cholesterol, HDL and LDL levels in women compared to men. The overall use of statin was significantly higher in men, whilst the intake of diuretic drugs was in women. Echocardiographic parameters were those estimated in patients with severe AS (Table 1).

**Table 1:** Demographical and clinical data of aortic stenosis patients.

	**Total**	**Men**	**Women**	***p*-value**
*n* (%)	202 (100)	123 (60.89)	79 (39.11)	
Age [mean (SD)]	71.66 (9.09)	69.88 (9.38)	74.41 (7.93)	<0.001
Weight [mean (SD)]	76.83 (14.33)	81.74 (12.59)	69.13 (13.55)	<0.001
Height [mean (SD)]	162.35 (8.78)	167.68 (5.52)	154.01 (5.99)	<0.001
Body surface [mean (SD)]	1.82 (0.19)	1.91 (0.15)	1.67 (0.16)	<0.001
DM, *n* (%)	61 (30.5)	38 (31.1)	23 (29.5)	0.927
HTA, *n* (%)	142 (71.0)	87 (71.3)	55 (70.5)	1.000
BAV, *n* (%)	85 (45.7)	55 (45.5)	30 (46.2)	1.000
**Drug medicines**				
ACEI, *n* (%)	51 (25.8)	30 (24.8)	21 (27.3)	0.824
ARB, *n* (%)	51 (25.8)	32 (26.4)	19 (24.7)	0.912
Spironolactone, *n* (%)	3 (1.5)	1 (0.8)	2 (2.6)	0.691
Eplerenone, *n* (%)	3 (1.5)	2 (1.7)	1 (1.3)	1.000
Diuretics, *n* (%)	112 (56.0)	60 (49.2)	52 (66.7)	0.022
β-blockers, *n* (%)	55 (27.5)	35 (28.7)	20 (25.6)	0.758
Statins, *n* (%)	129 (64.5)	91 (74.6)	38 (48.7)	<0.001
**Biochemical analyses**				
Total cholesterol [mean (SD)]	177.38 (40.67)	168.33 (36.89)	191.93 (42.45)	<0.001
Triglycerides (mg/dL) [mean (SD)]	112.15 (59.19)	113.04 (67.94)	110.75 (42.17)	0.798
HDL (mg/dL) [mean (SD)]	46.83 (12.92)	45.20 (12.16)	49.40 (13.74)	0.030
LDL (mg/dL) [mean (SD)]	107.75 (34.81)	100.13 (31.84)	119.72 (36.11)	<0.001
**Echocardiographic parameters**				
Max. gradient [mean (SD)]	79.19 (18.89)	78.57 (18.77)	80.17 (19.16)	0.566
Medium gradient [mean (SD)]	50.63 (13.06)	49.88 (12.81)	51.81 (13.44)	0.317
Valvular area echocardiography, cm^2^ [mean (SD)]	0.79 (0.47)	0.84 (0.59)	0.70 (0.20)	0.076
End-systolic volume (cc) [mean (SD)]	46.74 (31.49)	52.05 (34.57)	38.56 (24.12)	0.011
EF % [mean (SD)]	65.85 (13.12)	65.12 (13.35)	66.96 (12.76)	0.343

*N, sample size (biological replicates); SD, standard deviation; DM, diabetes mellitus; HTA, arterial hypertension; BAV, bicuspid aortic valve; ACEI, angiotensin converting enzyme inhibitors; ARB, angiotensin II receptor blockers; HDL, high density lipoproteins; LDL, low density lipoprotein; EF, ejection fraction*.

In addition, 36 patients (33.33% women) were recruited (cohort 2) to isolate VICs for *in vitro* experiments. No significant differences were found in this cohort except for body weight. Baseline clinical and demographical characteristics are summarized in Supplemental Table 1.

### Female Sex Is Associated With Lower Levels of Inflammatory and Oxidative Stress Markers in Human AVs

**Figure 1:**
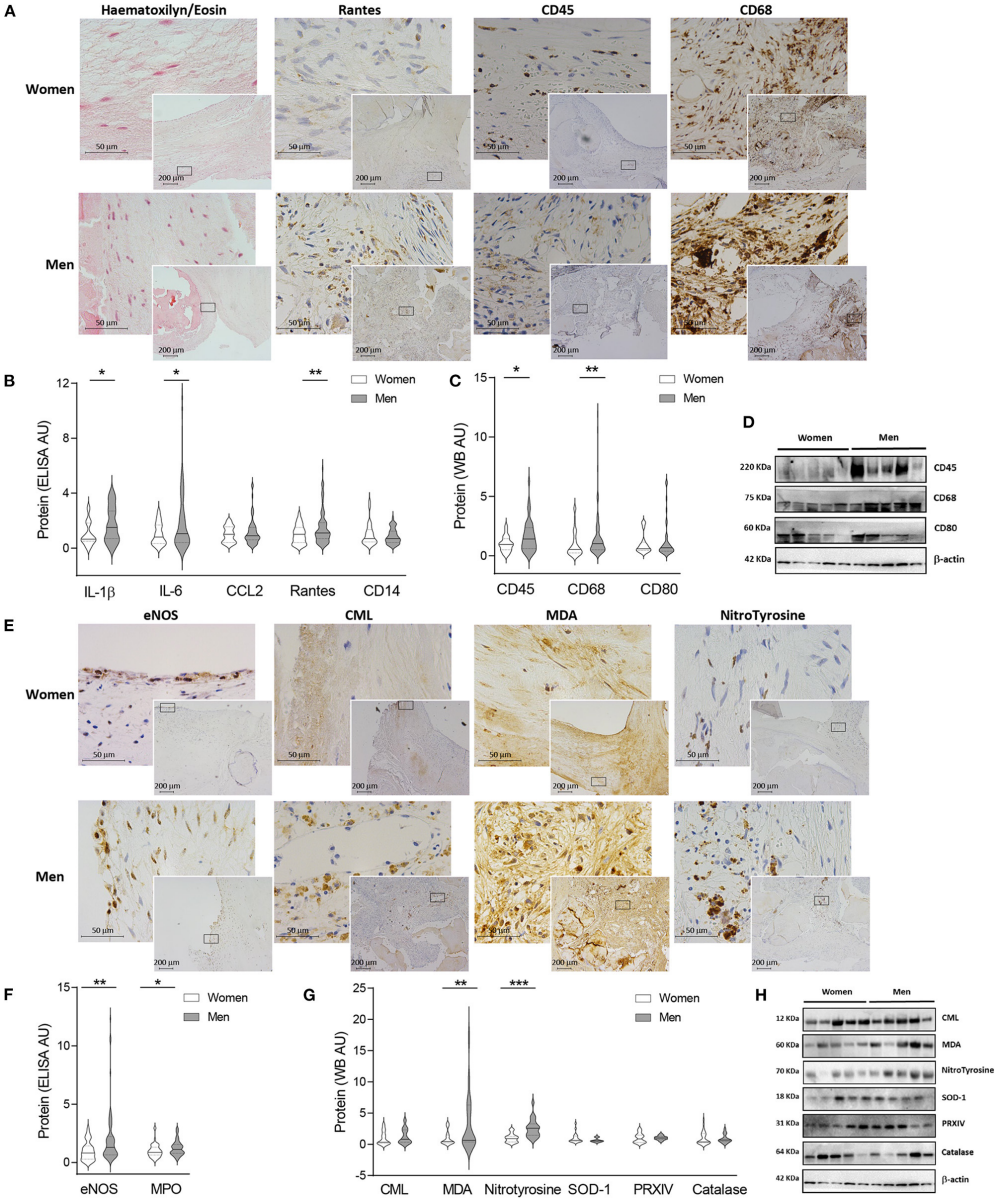
Sex differences in inflammation and oxidative stress markers in AVs from AS patients. Representative microphotographs of AV sections from AS patients stained for haematoxylin/eosin, and immunostained for Rantes, CD45 and CD68 (from left to right) in women and men AV tissue **(A)**. Protein expressions from different inflammation markers in tissue homogenates from AVs of AS patients measured by ELISA **(B)** and western blot **(C)**. Representative images for inflammatory markers measured by western blot **(D)**. Representative microphotographs of AV sections from AS patients immunostained for eNOS, CML, MDA, and Nitrotyrosine (from left to right) in women and men tissue **(E)**. Protein levels from oxidative stress markers in women and men aortic valve tissue measured by ELISA **(F)** and western blot **(G)**. Representative images of western blots for oxidative stress markers **(H)**. AS, aortic stenosis; AV, aortic valve; IL, interleukin; CCL2, C-C Motif Chemokine Ligand 2; eNOS, endothelial nitric oxide synthase; MPO, myeloperoxidase; CML, carboximethyl-lysine; MDA, malondialdehyde; SOD-1, superoxide dismutase-1; PRXIV, peroxiredoxin IV. The black solid line in the center of violin plots represent median and the dashed lines represent the interquartile range of each group of subjects in arbitrary units (AU). All data were normalized to women. Markers were measured by ELISA or western blot (normalized to stain free protein and β-actin). *N* = 22–65 for women and *N* = 36–81 for men. ^*^p < 0.05 vs. Women; ^*^p < 0.05, ^**^p < 0.01, ^***^p < 0.001, ^****^p < 0.0001.

Haematoxylin-eosin microphotographs suggest that AVs from women exhibited lower levels of infiltrating cells (Figure 1A). Complementary immunohistochemical analyses showed that AVs from women were less positive than men for inflammatory molecules such as Rantes, CD45 (leucocyte marker) and CD68 (macrophage marker) (Figure 1A and Supplemental Figures 2A,B). At protein level, women had lower levels of IL-1β(5.95 ± 1.1 vs. 10.23 ± 1.4 pg/ml; 1.00 ± 0.2 vs. 1.72 ± 0.2 fold-change; *p* = 0.0218), IL-6 (8.49 ± 1.1 vs. 18.11 ± 2.8pg/ml; 1.00 ± 0.1 vs. 2.13 ± 0.3 fold-change; *p* = 0.0428), Rantes (13.59 ± 1.1 vs. 20.62 ± 1.8 pg/ml; 1.00 ± 0.1 vs. 1.52 ± 0.1 fold-change *p* = 0.0091) (Figure 1B); CD45 (0.15 ± 0.02 vs. 0.23 ± 0.02 A.U.; 1.00 ± 0.1 vs. 1.6 ± 0.17 fold-change *p* = 0.0385) and CD68 (0.096 ± 0.02 vs. 0.163 ± 0.03 A.U.; 1.00 ± 0.16 vs. 1.71 ± 0.27 fold-change; *p* = 0.0027) than men (Figure 1C). No differences between women and men AVs were found for CCL2 (60.23 ± 5.6 vs. 72.23 ± 8 pg/ml; 1.00 ± 0.1 vs. 1.2 ± 0.1 fold-change), CD14 (monocyte marker) (614.7 ± 103 vs. 529 ± 50 pg/ml; 1.00 ± 0.2 vs. 0.86 ± 0.1 fold-change) (Figure 1B) and CD80 (0.021 ± 0.004 vs. 0.028 ± 0.006 A.U.; 1.00 ± 0.2 vs. 1.34 ± 0.29 fold-change) expressions (Figure 1C). Importantly, after adjusting for age, lipid profile and statins treatment, women AVs exhibited lower inflammatory markers IL-6 (OR = −33.08, *p* = 0.007), Rantes (OR = −8.73, *p* = 0.001), CD45 (OR = −0.1, *p* = 0.012) and CD68 (OR = −0.1, *p* = 0.010) (Supplementary Table 2). Representative blots for inflammatory markers are shown in Figure 1D.

The analysis of oxidative stress markers hint an overall enhanced oxidative stress in immunohistological AVs preparations for eNOS, carboxymethyl-lysine, malondialdehyde and nitrotyrosine in AVs from men (Figure 1E). These findings were confirmed using ELISA/WB approaches. Women-derived AVs exhibited decreased oxidative stress markers such as eNOS (114.6 ± 13.9 vs. 238.6 ± 33.8 pg/ml; 1.00 ± 0.1 vs. 2.08 ± 0.3 fold-change; *p* = 0.0011), myeloperoxidase (1,286 ± 104.7 vs. 1,594 ± 102 pg/ml; 1.00 ± 0.1 vs. 1.24 ± 0.1 fold-change; *p* = 0.0184) (Figure 1F), malondialdehyde (3.99 ± 0.8 vs. 14.23 ± 3.3 A.U.; 1.00 ± 0.2 vs. 3.56 ± 0.8 fold-change; *p* = 0.0092) or nitrotyrosine (0.242 ± 0.04 vs. 0.622 ± 0.1 A.U.; 1.00 ± 0.2 vs. 2.58 ± 0.3 fold-change; *p* = 0.0002) as compared to men's (Figure 1G). Superoxide dismutase-1 was slightly higher in women although it did not reach statistical significance (2.27 ± 0.4 vs. 1.46 ± 0.2 A.U.; 1.00 ± 0.2 vs. 0.64 ± 0.1 fold-change) (Figure 1G). However, similar amounts of carboxymethyl-lysine (0.91 ± 0.2 vs. 1.20 ± 0.2 A.U.; 1.00 ± 0.2 vs. 1.31 ± 0.3 fold-change), peroxiredoxin-IV (0.86 ± 0.2 vs. 0.88 ± 0.1 A.U.; 1.00 ± 0.2 vs. 1.03 ± 0.19 fold-change) and catalase (1.67 ± 0.3 vs. 1.48 ± 0.3 A.U.; 1.00 ± 0.2 vs. 0.89 ± 0.2 fold-change) were reported in AVs from both sexes (Figure 1G). Notably, women AVs exhibited lower oxidative stress markers eNOS (OR = −176.43, *p* = 0.0004) and nitrotyrosine (OR = −0.39, *p* = 0.002) after adjusting for age, lipid profile and statins treatment (Supplementary Table 2). Representative blots for oxidative stress markers are shown in Figure 1H.

### Women-Derived AVs Present Less Fibrosis but More ECM Remodeling Than Men's

**Figure 2:**
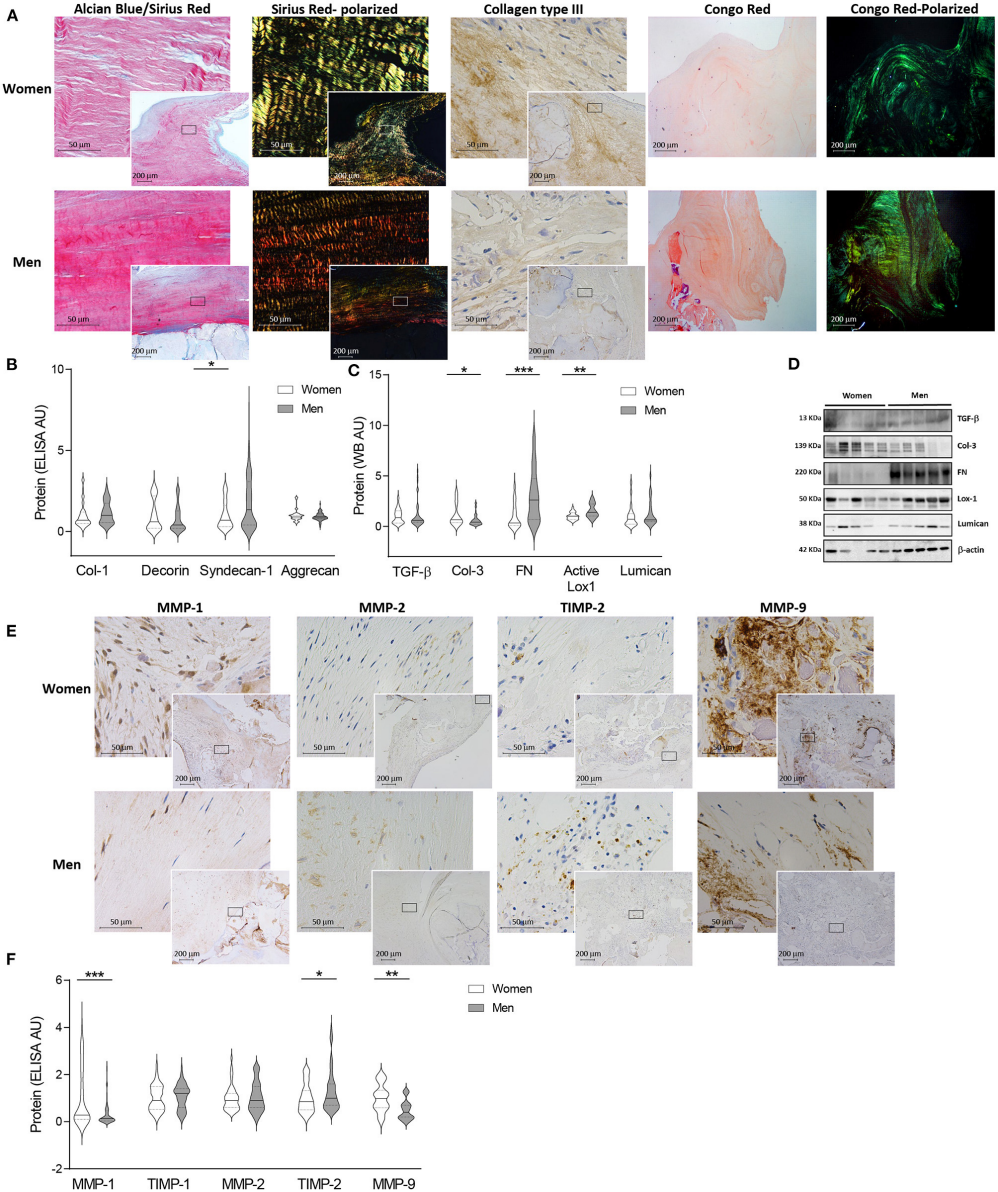
Fibrosis and ECM remodeling markers in women and men AV tissue. Representative microphotographs of AV sections from AS patients stained with alcian blue and Sirius red (AB/SR) double staining, sirius red (SR) with polarized light, collagen type III immunohistochemistry and Congo red with bright field and polarized **(A)**. Protein levels of several fibrosis and ECM remodeling markers from human AV tissue measured by ELISA **(B)** and western blot **(C)**. Representative western blot images for fibrosis markers **(D)**. Representative immunostainings for MMP-1, MMP-2, TIMP-2 and MMP-9 in women and men valve tissue **(E)**. ECM remodeling markers at protein level in human tissue homogenates from AS patients **(F)**. AS, aortic stenosis; AV, aortic valve; ECM, extracellular matrix; TGF-β, transforming Growth Factor-β1; Col, collagen; FN, fibronectin; Lox-1, lysyl oxidase-1; MMP, matrix metalloproteinase; TIMP, metalloproteinase inhibitor. The black solid line in the center of violin plots represent median and the dashed lines represent the interquartile range of each group of subjects in arbitrary units (AU). All data were normalized to women. Markers were measured by ELISA or western blot (normalized to stain free protein and β-actin). *N* = 24–65 for women and *N* = 23–79 for men. ^*^p < 0.05 vs. Women; ^*^p < 0.05, ^**^p < 0.01, ^***^p < 0.001, ^****^p < 0.0001.

ECM content and distribution were analyzed in AVs from women and men. As shown in double alcian blue-sirius red staining, thicker collagen fibers were presented in AVs from men as compared to women's, whilst thinner fibers were reported in women's AVs (Figure 2A and Supplementary Figures 2C,D). In addition, Congo red staining suggested more β-amyloid deposits in AVs from men (Figure 2A), although the digital image analysis of 44 preparations (45.45% women) did not reach the statistical significance (Supplementary Figure 2E). These deposits were further validated by β-amyloid immunohistochemistry (Supplementary Figure 3A). In addition, elastic fibers disarray and fragmentation were studied in both sexes for further anatomopathological characterization. Elastic fibers were disrupted and disarrayed in both sexes, although more lax fibers were exhibited in women AVs as compared to men (Supplementary Figure 3A).

The content of fibrosis markers and proteoglycans was quantified in AVs from women and men (Figures 2B,C). AVs from women presented similar levels of collagen type I (614.05 ± 63.8 vs. 709.03 ± 50.4 pg/ml; 1.00 ± 0.1 vs. 1.16 ± 0.1 fold-change) (Figure 2B) and enhanced levels of collagen type III (1.582 ± 0.3 vs. 0.878 ± 0.1 A.U.; 1.00 ± 0.2 vs. 0.56 ± 0.1 fold-change; *p* = 0.0322) (Figure 2C). However, AVs from women exhibited lower fibronectin (0.55 ± 0.2 vs. 1.69 ± 0.3 A.U.; 1.00 ± 0.3 vs. 3.07 ± 0.5 fold-change; *p* = 0.0009), active lysyl oxidase-1 (0.68 ± 0.1 vs. 1.05 ± 0.1 A.U.; 1.00 ± 0.1 vs. 1.54 ± 0.1 fold-change; *p* = 0.0045) (Figure 2C) and syndecan-1 (347.8 ± 40.2 vs. 637 ± 70.2 pg/ml; 1.00 ± 0.1 vs. 1.83 ± 0.2 fold-change; *p* = 0.0202) (Figure 2B) as compared to AVs from men. No changes were reported among women and men in decorin (448.55 ± 69.9 vs. 363.88 ± 48.8 pg/ml; 1.00 ± 0.2 vs. 0.81 ± 0.1 fold-change) and aggrecan (1188.1 ± 70.3 vs. 1085.7 ± 38.4 pg/ml; 1.00 ± 0.1 vs. 0.91 ± 0.03 fold-change) expressions (Figure 2B) as well as TGF-β(0.21 ± 0.03 vs. 0.25 ± 0.1 A.U.; 1.00 ± 0.2 vs. 1.23 ± 0.3 fold-change) and lumican (0.58 ± 0.1 vs. 0.76 ± 0.2 A.U.; 1.00 ± 0.3 vs. 1.31 ± 0.3 fold-change) (Figure 2C). Representative blots for fibrosis markers are displayed in Figure 2D. No changes in TGF-β, Collagen type I, III, decorin and lumican transcripts were found between sexes (Supplementary Figure 3B). Concerning fibrosis markers, women AVs exhibited lower fibronectin (OR = −23.69, *p* = 0.002) and syndecan-1 (OR = −234.45, *p* = 0.039), whereas collagen type III content was higher (OR = 0.66, *p* = 0.024) after adjusting for age, lipid profile and statins treatment (Supplementary Table 2).

We next investigated the expression of molecules involved in ECM degradation including MMPs and their inhibitors (TIMPs). Immunohistochemical analyses suggested an overall enhanced expression of MMPs and decreased TIMPs in AVs from women (Figure 2E). These findings were further confirmed using quantitative analyses (Figure 2F). Accordingly, AVs from women presented increased levels of MMP-1 (277.4 ± 44.1 vs. 89.2 ± 15 pg/ml; 1.00 ± 0.2 vs. 0.32 ± 0.1 fold-change; *p* = 0.0003) and MMP-9 (184.55 ± 22.8 vs. 90.47 ± 12.1 pg/ml; 1.00 ± 0.1 vs. 0.49 ± 0.07 fold-change; *p* = 0.0011) and decreased TIMP-2 (479.5 ± 37.9 vs. 634.1 ± 48.1 pg/ml; 1.00 ± 0.08 vs. 1.32 ± 0.1 fold-change; *p* = 0.0192). No changes in TIMP-1 (874.7 ± 68.1 vs. 933.0 ± 57.6pg/ml; 1.00 ± 0.08 vs. 1.07 ± 0.07 fold-change) and MMP-2 (1.76 ± 0.14 vs. 1.85 ± 0.13ng/ml; 1.00 ± 0.08 vs. 1.05 ± 0.08 fold-change) were found (Figure 2F). At the transcription level, a significantly increase in MMP-9 expression (*p* = 0.0410) was found in males without changes in MMP-1, TIMP-1, MMP-2 or TIMP-2 (Supplementary Figure 3C). Further MMP activity analyses revealed a significantly enhanced activity of MMP-1 (1.67 ± 0.1 vs. 1.24 ± 0.1 A.U.; 1.00 ± 0.1 vs. 0.74 ± 0.1 fold-change; *p* = 0.0148) in women as compared to men AVs (Supplementary Figure 3D). No major changes either in MMP-2 (1.49 ± 0.13 vs. 1.25 ± 0.08 A.U.; 1.00 ± 0.1 vs. 0.89 ± 0.1 fold-change) or MMP-9 activities (1.59 ± 0.27 vs. 1.22 ± 0.16 A.U.; 1.00 ± 0.2 vs. 0.77 ± 0.1 fold-change) were reported. Interestingly, women AVs exhibited higher MMP-1 (OR = 299.65, *p* = 0.0002), MMP-9 (OR = 84.58, *p* = 0.003) and MMP-1 activity (OR = 0.51, *p* = 0.024) and lower TIMP-2 (OR = −164.34, *p* = 0.023) after adjusting for age, lipid profile and statins treatment (Supplementary Table 2). Representative zymography for MMP-9, 2 and 1 activities are shown in Supplementary Figure 3E.

### AVs From Women Exhibited Decreased Calcification and Osteogenic Differentiation

**Figure 3:**
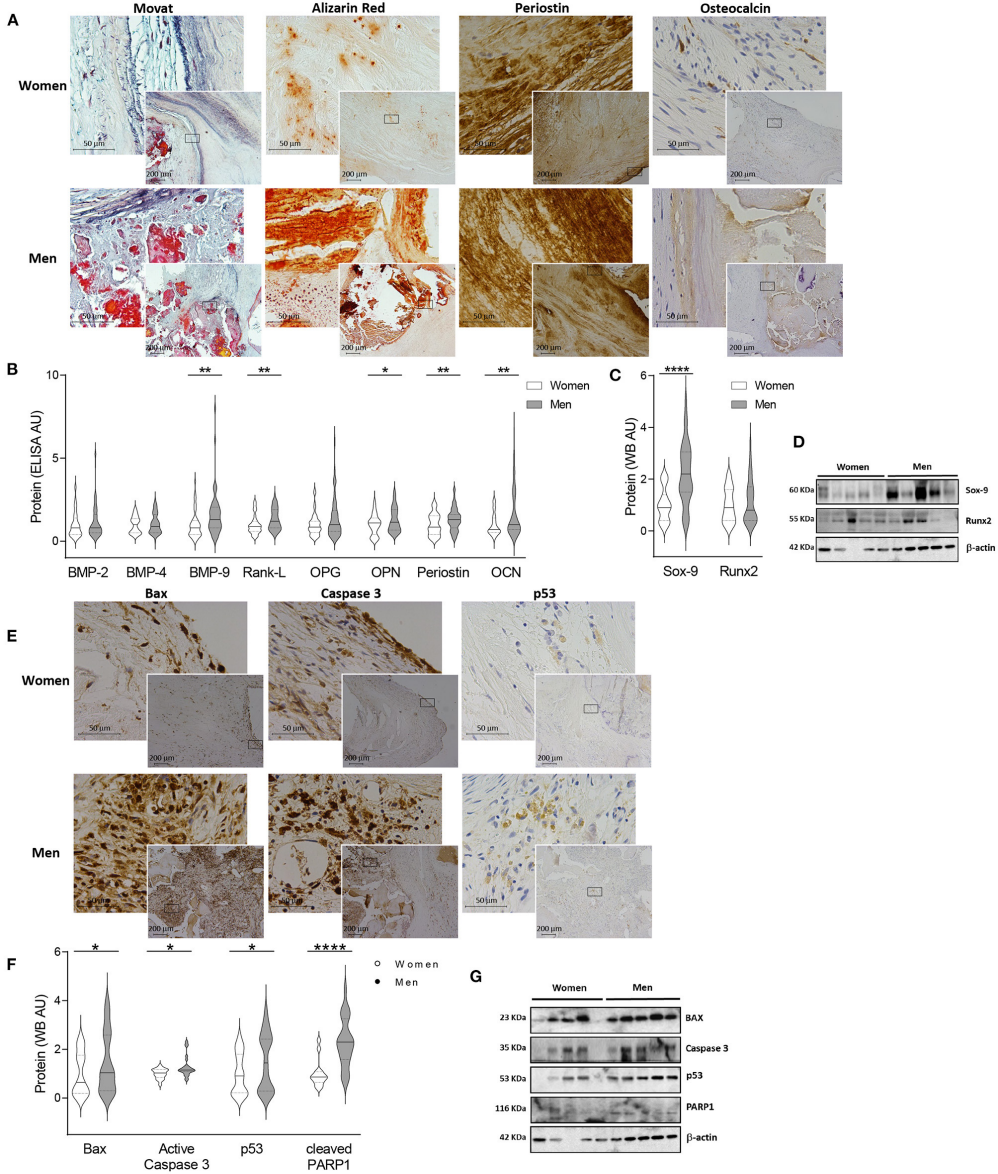
Sexual dimorphism in calcification and apoptosis markers in human AVs. Representative images for Movat, Alizarin red (AR), periostin and osteocalcin staining in women and men AVs slides **(A)**. Protein expression of calcification markers in women and men AV tissues measured by ELISA **(B)** and western blot **(C)**. Representative western blot images for calcification markers **(D)**. Representative microphotographs of Bax, Caspase 3 and p53 immunostainings in women and men AV tissues **(E)**. Protein expression of apoptosis markers in women and men tissue homogenates **(F)**. Representative blots for apoptosis markers **(G)**. AS, aortic stenosis; AV, aortic valve; BMP, bone morphogenetic protein; Rank-L, Receptor activator of NF-kappa B ligand; OPG, osteoprotegerin; OPN, osteopontin; OCN, osteocalcin; Sox-9, SRY (sex-determining region Y)-box 9; Runx2, runt-related transcription factor 2; Bax, BCL-2 associated protein; PARP1, Poly [ADP-ribose] polymerase 1. The black solid line in the center of violin plots represent median and the dashed lines represent the interquartile range of each group of subjects in arbitrary units (AU). All data were normalized to women. Markers were measured by ELISA or western blot (normalized to stain free protein and β-actin). *N* = 19–56 for women and *N* = 33–79 for men. ^*^p < 0.05 vs. Women; ^*^p < 0.05, ^**^p < 0.01, ^***^p < 0.001, ^****^p < 0.0001.

Movat pentachrome staining evidenced a higher presence of mature mineralized and non-mineralized bone, seen as bright yellow and bright red, respectively, in men-derived AVs (Figure 3A and Supplementary Figures 2F,G). In order to specifically analyse AV calcification, Alizarin red staining was additionally performed, showing greater calcium deposits in AVs from men (Supplementary Figure 2H). These findings were paralleled with an increase in late osteoblast-differentiation markers such as periostin and osteocalcin assessed by immunohistochemistry in AVs from men (Figure 3A).

In line with these results, the quantification of osteogenic markers showed lesser levels of BMP-9 (39.92 ± 5 vs. 65.89 ± 8 pg/ml; 1.00 ± 0.1 vs. 1.65 ± 0.2 fold-change; *p* = 0.0015), Rank-L (850.58 ± 68.1 vs. 1120.83 ± 68.7 pg/ml; 1.00 ± 0.1 vs. 1.32 ± 0.1 fold-change; *p* = 0.0026), osteopontin (1364.6 ± 117 vs. 1,759 ± 127 pg/ml; 1.00 ± 0.1 vs. 1.29 ± 0.1 fold-change; *p* = 0.0455), periostin (1324.9 ± 118 vs. 1737.9 ± 114 pg/ml; 1.00 ± 0.1 vs. 1.31 ± 0.1 fold-change; *p* = 0.0099), osteocalcin (354.33 ± 31.3 vs. 607.9 ± 60.8 pg/ml; 1.00 ± 0.1 vs. 1.72 ± 0.2 fold-change; *p* = 0.0018) (Figure 3B) and Sox-9 (0.72 ± 0.1 vs. 1.58 ± 0.1 A.U.; 1.00 ± 0.1 vs. 2.2 ± 0.2 fold-change; *p* < 0.0001) (Figure 3C) in AVs from women as compared to men. Male and female AV show comparable expression for BMP-2 (144.76 ± 15.8 vs. 173.59 ± 17.9 pg/ml; 1.00 ± 0.1 vs. 1.2 ± 0.1 fold-change; p = 0.1899), BMP-4 (128.9 ± 10.4 vs. 137.4 ± 9.4 pg/ml; 1.00 ± 0.1 vs. 1.1 ± 0.1 fold-change; p = 0.3552), osteoprotegerin (614.39 ± 55.8 vs. 891.22 ± 89.3 pg/ml; 1.00 ± 0.1 vs. 1.45 ± 0.1 fold-change; p = 0.0595) (Figure 3B) and Runx2 (0.95 ± 0.1 vs. 1.09 ± 0.1 A.U.; 1.00 ± 0.1 vs. 1.14 ± 0.1 fold-change; p = 0.3420) (Figure 3C). Importantly, women AVs exhibited reduced BMP-9 (OR = −19.84, *p* = 0.06), osteopontin (OR = −439.17, *p* = 0.028), osteocalcin (OR = −171.61, *p* = 0.059) and Sox-9 (OR = −0.7, *p* = 0.006) after adjusting for age, lipid profile and statins treatment (Supplementary Table 2). Representative blots for selected osteogenic markers are shown in Figure 3D. At the mRNA levels, osteopontin (*p* = 0.0001), periostin (*p* = 0.0065) and Sox-9 (*p* < 0.0001) were found increased in men valves with a rise tendency for BMP-2, BMP-4 and Runx2 (Supplementary Figure 2F).

### Apoptotic Pathways Are Less Activated in Women-Derived AVs

AVs from men exhibited increased immunostaining for the pro-apoptotic markers Bax, Caspase-3 and p53 as compared with women's AVs (Figure 3E). To further validate such results, these pro-apoptotic markers were quantified and were found diminished in women AV as compared to men's: Bax (0.69 ± 0.1 vs. 1.01 ± 0.1 A.U.; 1.00 ± 0.2 vs. 1.46 ± 0.2 fold-change; *p* = 0.0362), Caspase-3 (0.84 ± 0.04 vs. 1.08 ± 0.1 A.U.; 1.00 ± 0.05 vs. 1.29 ± 0.1 fold-change *p* = 0.0127), p53 (0.38 ± 0.1 vs. 0.57 ± 0.1 A.U.; 1.00 ± 0.1 vs. 1.49 ± 0.2 fold-change; *p* = 0.0147) and cleaved PARP1 (0.23 ± 0.04 vs. 0.52 ± 0.05 A.U.; 1.00 ± 0.2 vs. 2.29 ± 0.2 fold-change; *p* < 0.0001) (Figure 3F). After adjusting for age, lipid profile and statins treatment only Bax was decreased in women AVs as compared to men's (OR = −0.49, *p* = 0.02) (Supplementary Table 2). Representative blots for apoptosis markers are displayed in Figure 3G.

### Association Among Collagen Type III and Molecules Involved in Pathophysiological Pathways in AS Patients

Since previous investigations have described sex-differences in AV collagen content between women and men, we next investigated whether collagen content of AVs could be related with AS pathophysiology. Thus, correlation analyses were performed between AV collagen type III expression and parameters assessing inflammation, oxidative stress, calcification and clinical features of AS. In the whole cohort, collagen type III expression negatively correlated with parameters showing inflammation (Rantes; *r* = −0.3198, *p* = 0.0062), oxidative stress (myeloperoxidase; *r* = −0.275, *p* = 0.0402) and calcification (BMP-9; *r* = −0.4183, *p* = 0.0072; osteopontin; *r* = −0.2815, *p* = 0.0356; periostin; *r* = −0.3684, *p* = 0.0061).

### *In vitro* Analyses in Human Aortic VICs

**Figure 4:**
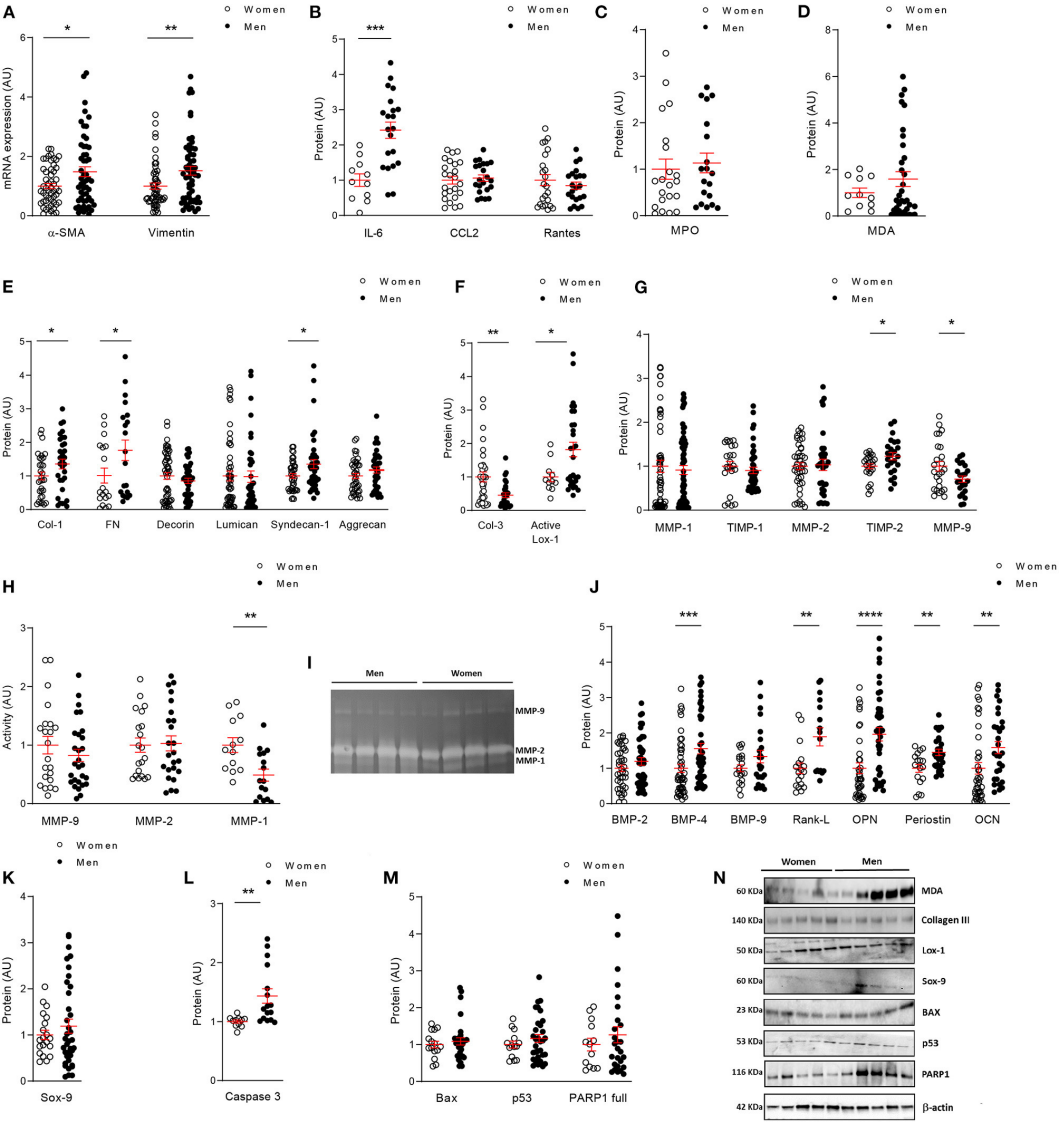
*In vitro* comparative analysis between human VICs from women and men. mRNA levels of VICs activation markers **(A)** in women and men VICs. Protein expressions of inflammatory markers by ELISA **(B)**, oxidative stress markers by ELISA **(C)** or western blot **(D)**; and fibrosis markers analyzed by ELISA **(E)** or western blot **(F)** in VICs from male and female human AVs. Comparison of ECM remodeling protein levels **(G)** and activity **(H)** in women and men VICs. Representative zymograme of MMP-9,−2 and−1 **(I)**. Levels of calcification markers measured by ELISA **(J)** or western blot **(K)**; and apoptosis markers studied by ELISA **(L)** or western blot **(M)** in human VICs. Representative western blots for oxidative stress, fibrosis, calcification and apoptotic markers **(N)**. ECM, extracellular matrix; α-SMA, alpha smooth muscle actin; IL, interleukin; CCL2, C-C Motif Chemokine Ligand 2; MPO, myeloperoxidase; MDA, malondialdehyde; Col, collagen; FN, fibronectin; Lox-1, lysyl-oxidase-1; MMP, matrix metalloproteinase; TIMP, metalloproteinase inhibitor; BMP, bone morphogenetic protein; Rank-L, Receptor activator of NF-kappa B ligand; OPN, osteopontin; OCN, osteocalcin; Sox-9, SRY (sex-determining region Y)-box 9; BAX, BCL-2-associated protein; PARP1, Poly [ADP-ribose] polymerase 1. Gene expression data were normalized to HPRT, β-actin and GADPH. Dot plots represent mean and standard error of the mean (SEM) of each group of subjects (women *n* = 12 and men *n* = 24, VICs from each patient have 3–8 replicates) in arbitrary units (AU). All data were normalized to women. Protein markers were measured by ELISA or western blot (normalized to stain free protein and β-actin). ^*^p < 0.05 vs. Women; ^*^p < 0.05, ^**^p < 0.01, ^***^p < 0.001, ^****^p < 0.0001.

We next examined if the reported sex-related differences in whole AVs were kept in cultured VICs isolated from male or female-derived AVs (*n* = 36, 33.33% women donors). Our results showed that VICs from women presented lower expression of myofibroblast activation markers (α-SMA (1.63 ± 0.2 vs. 2.41 ± 0.27 A.U.; 1.00 ± 0.09 vs. 1.48 ± 0.16 fold-change; *p* = 0.0399) and vimentin (4.9 ± 0.5 vs. 7.5 ± 0.7 A.U.; 1.00 ± 0.1 vs. 1.52 ± 0.15 fold-change; *p* = 0.0092)) (Figure 4A) and IL-6 (14,838 ± 2,525 vs. 35,891 ± 3,343 pg/ml, 1.00 ± 0.17 vs. 2.42 ± 0.23 fold-change; *p* = 0.0001), without changes in CCL2 (562 ± 60 vs. 596 ± 50 pg/ml; 1.00 ± 0.11 vs. 1.06 ± 0.09 fold-change) and Rantes (3,713 ± 569 vs. 3,136 ± 369 pg/ml; 1.00 ± 0.15 vs. 0.84 ± 0.1 fold-change) (Figure 4B). At the mRNA levels, the expression of IL-6 and CCL2 (*p* = 0.0136) was augmented in male VICs (Supplementary Figure 4A). A slightly, but not significant, decrease in oxidative stress markers myeloperoxidase (6,095 ± 1,292 vs. 6,913 ± 1,262 pg/ml; 1.00 ± 0.21 vs. 1.13 ± 0.21 fold-change) (Figure 4C) and malondialdehyde (0.084 ± 0.02 vs. 0.133 ± 0.03 A.U.; 1.00 ± 0.19 vs. 1.59 ± 0.31 fold-change) (Figure 4D) was found in women VICs as compared to men.

Regarding the expression of fibrosis markers, VICs isolated from women secreted lower levels of collagen type I (30,570 ± 3,574 vs. 41,466 ± 4,478 pg/ml; 1.00 ± 0.12 vs. 1.36 ± 0.15 fold-change; *p* = 0.0314), fibronectin (4,868 ± 1,041 vs. 8,481 ± 1,433 ng/ml; 1.00 ± 0.22 vs. 1.74 ± 0.3 fold-change; *p* = 0.0210), Syndecan-1 (491.1 ± 34.4 vs. 664.6 ± 65.3 pg/ml; 1.00 ± 0.07 vs. 1.35 ± 0.13 fold-change; *p* = 0.0316) (Figure 4E). No changes were found for decorin (7,104 ± 698 vs. 6,074 ± 543 pg/ml; 1.00 ± 0.1 vs. 0.86 ± 0.08 fold-change), lumican (49,017 ± 7,086 vs. 48,096 ± 8,167 pg/ml; 1.00 ± 0.14 vs. 0.98 ± 0.17 fold-change) or aggrecan (929 ± 71.4 vs. 1,093 ± 83.9 pg/ml; 1.00 ± 0.08 vs. 1.18 ± 0.09 fold-change) (Figure 4E). Moreover, VICs expressed less active lysyl oxidase-1 (1.49 ± 0.2 vs. 2.72 ± 0.3 A.U.; 1.00 ± 0.13 vs. 1.82 ± 0.22 fold-change; *p* = 0.0277) (Figure 4F). Interestingly, collagen type III levels were enhanced in female VICs as compared to male VICs (0.03 ± 0.004 vs. 0.013 ± 0.002 A.U.; 1.00 ± 0.15 vs. 0.47 ± 0.07 fold-change; *p* = 0.0013) (Figure 4F). At the mRNA level, collagen type I was consistently enhanced in men (*p* = 0.0175) and a trend was reported for fibronectin and decorin (Supplementary Figure 4B).

We assessed the expression and activity of matrix-degrading enzymes potentially involved in ECM homeostasis in VICs from women and men. Our results showed increased levels of MMP-9 (19,663 ± 2,135 vs. 13,874 ± 1,349 pg/ml; 1.00 ± 0.11 vs. 0.71 ± 0.07 fold-change; *p* = 0.0164) and lower levels of TIMP-2 (65,793 ± 3,649 vs. 80,749 ± 5,321 pg/ml; 1.00 ± 0.06 vs. 1.23 ± 0.08 fold-change; *p* = 0.0217) in women VICs as compared to men VICs (Figure 4G). Similar levels for MMP-1 (1,894 ± 257 vs. 1,729 ± 190 pg/ml; 1.00 ± 0.14 vs. 0.91 ± 0.10 fold-change), TIMP-1 (151,912 ± 16,032 vs. 137,301 ± 10,946 pg/ml; 1.00 ± 0.11 vs. 0.90 ± 0.07 fold-change) and MMP-2 (288 ± 22.6 vs. 297 ± 36.6 ng/ml; 1.00 ± 0.08 vs. 1.03 ± 0.13 fold-change) (Figure 4G) were found in VICs from both sexes. Regarding MMPs activity we found a significant decrease in MMP-1 (*p* = 0.0018), a decline tendency in MMP-9 and no changes in MMP-2 in men VICs (Figure 4H). Representative zymograme of MMP-9, 2 and 1 activities is shown in Figure 4I.

Moreover, women-derived VICs secreted lower amounts of BMP-4 (90.14 ± 10.4 vs. 140.33 ± 13.2 pg/ml; 1.00 ± 0.12 vs. 1.56 ± 0.15 fold-change; *p* = 0.0006), Rank-L (33,386 ± 4,880 vs. 63,046 ± 8,305 pg/ml; 1.00 ± 0.14 vs. 1.89 ± 0.25 fold-change; *p* = 0.0045), osteopontin (361.2 ± 54.5 vs. 708.7 ± 67.4 pg/ml; 1.00 ± 0.15 vs. 1.96 ± 0.19 fold-change; *p* < 0.0001), periostin (48,250 ± 5,284 vs. 69,904 ± 4,204 pg/ml; 1.00 ± 0.11 vs. 1.45 ± 0.09 fold-change; *p* = 0.0016) and osteocalcin (3,993 ± 641 vs. 6,324 ± 676 pg/ml; 1.00 ± 0.16 vs. 1.58 ± 0.17 fold-change; *p* = 0.0015) and a tendency for BMP-2 (218 ± 19 vs. 260 ± 23 pg/ml; 1.00 ± 0.09 vs. 1.2 ± 0.11 fold-change), BMP-9 (40.5 ± 3.8 vs. 53.9 ± 6.9 pg/ml; 1.00 ± 0.09 vs. 1.33 ± 0.17 fold-change) (Figure 4J); and Sox-9 (0.025 ± 0.002 vs. 0.03 ± 0.003 A.U.; 1.00 ± 0.1 vs. 1.19 ± 0.15 fold-change) (Figure 4K), all that compared to men-derived VICs. BMP-2 (*p* = 0.0163), BMP-4 (*p* < 0.0001), osteopontin (*p* = 0.0015), periostin (*p* = 0.0001), Sox-9 (*p* = 0.0003) and Runx2 (*p* < 0.0001) gene expression was enhanced in VICs isolated from men valves (Supplementary Figure 4C).

Finally, analyses of pro-apoptotic markers showed a decrease in caspase-3 (659.9 ± 17 vs. 944.5 ± 77.2 pg/mg; 1.00 ± 0.03 vs. 1.43 ± 0.12 fold-change; *p* = 0.0011) (Figure 4L) in women VICs and a tendency in p53 (0.059 ± 0.005 vs. 0.068 ± 0.006 A.U.; 1.00 ± 0.09 vs. 1.16 ± 0.11 fold-change) and PARP1 (0.09 ± 0.01 vs. 0.114 ± 0.02 A.U.; 1.00 ± 0.17 vs. 1.27 ± 0.22 fold-change). No changes in Bax levels were reported (0.038 ± 0.003 vs. 0.041 ± 0.003 A.U.; 1.00 ± 0.09 vs. 1.09 ± 0.09 fold-change) (Figure 4M). Representative blots for in vitro markers are shown in Figure 4N.

## Discussion

**Figure 5:**
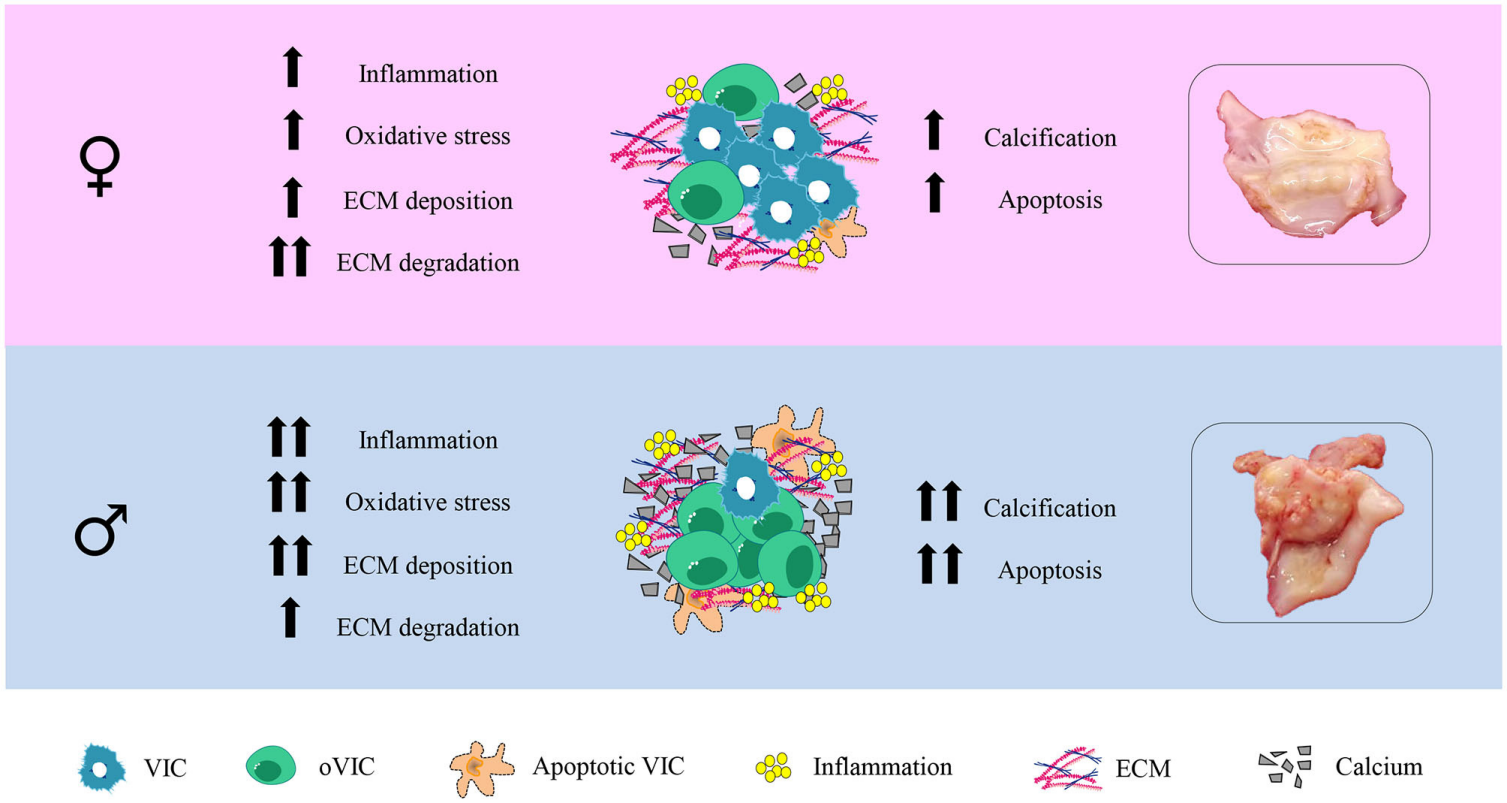
Graphical abstract.

Our study characterizes for the first time the sex-related histological and molecular expression patterns of inflammatory, oxidative stress, fibrosis, ECM remodeling, calcification and apoptosis markers in AVs. Such sex-specific profiles were further validated in isolated VICs from men and women with AS. Our data demonstrate that AVs from women exhibit less inflammation, oxidative stress, apoptosis and calcification than those from men for the same hemodynamic AS severity (Figure 5). The odds of having enhanced inflammation, oxidative stress, apoptosis and calcification in men AVs remained significant still when adjusting for age, lipid profile and statins treatment. Noteworthy, we firstly demonstrate that collagen type III content and overall ECM degradation markers were enhanced in AVs from women as compared to men, even after adjusting for the confounder factors. These results in AVs were further validated on *in vitro* cultured VICs from both sexes. Overall, the basal characterization of VICs confirmed that pro-fibro/inflammatory, pro-osteogenic and pro-apoptotic profiles were reduced in women-derived VICs, accompanied by enhanced ECM remodeling driven by higher MMP-1 and lower TIMP-2. These molecular findings might, therefore, be clinically relevant to the pathogenesis and outcomes of AS in a sex-dependent manner.

Accumulating evidences suggest that a number of pathophysiological mechanisms may contribute to the sex-related differences in hemodynamic severity in patients with AS (9, 11, 15). Inflammation and oxidative stress are closely related processes, linked with a number of chronic diseases including AS (16). Whether inflammation and oxidative stress promote AV remodeling and mineralization has not been demonstrated yet, although the inflammatory burden and the oxidative status seem to correlate with that of calcification (17–20). Our analyses revealed diminished inflammatory and oxidative stress markers in women-derived AVs, which could explain the lower calcification load reported in the latter. Accordingly, VICs were *per se* a source of pro-inflammatory cytokines which could lead to an enhanced pro-inflammatory and pro-oxidant status mediating and exacerbating calcification cues (21). The underlying mechanism of increased eNOS in men likely involves an increased production of H_2_O_2_. NOS uncoupling has been associated with the development of AS (20). Moreover, in agreement with our *in vitro* results, a recent publication suggested that VICs are an additional source of eNOS expression in the valve (22), being greater expressed in male porcine VICs than in the female counterparts (6). As a result, accumulation of oxidation products during early valve lesions may enhance inflammation, apoptosis and MMPs. Indeed, inflammation seems to drive different sex-related AS phenotypes. Transcriptomic analyses revealed overrepresented inflammation pathways in male porcine VICs (6). Recently, Parra-Izquierdo et al. (21, 23) have described a link among inflammatory factors, the expression of hypoxic inducible factors and an enhanced pro-calcific profile in human VICs isolated from men donors. Thus, it is interesting to speculate that inflammation and oxidative stress cues are overactivated in men AVs, mechanistically contributing to the increased calcification load reported in men by us and others (9, 11, 24).

Previous studies have shown that collagen fibers are more abundant in women than in men (11). In our cohort, we have analyzed more in depth the specific expression of collagen types, namely collagen type I and collagen type III. Our results show that AVs from women presented more collagen type III content, with no differences in collagen type I compared to men. It is important to point out the different technical approaches used; in our study, specifically collagen type I and III were quantified by quantitative or semi-quantitative methods in whole AV tissues, whereas in earlier studies total collagen fibers and dense connective tissue were quantified histologically (24). Interestingly, primary VICs isolated from women AVs exhibited less collagen type I expression and secretion but higher collagen type III expression. Negative associations among collagen type III expression and markers of inflammation, oxidative stress and calcification might suggest its involvement in the pathophysiology of AS in women. Interestingly, the increase in collagen type III expression in female AVs was accompanied by diminished expression of other fibrotic molecules such as fibronectin, syndecan-1 and active lysyl oxidase-1. Fibronectin, a protein secreted in response to damage (25) could bind to collagens or proteoglycans conferring stiffness to the AV (26). The increase in syndecan-1 observed in men's AVs could be related to the higher degree of differentiation observed in male isolated VICs (27). Moreover, enhanced lysyl oxidase-1 activity found in men AVs has been previously associated with inflammation, fibrosis and calcification (28–30).

The balance between ECM synthesis and degradation is involved in the structural integrity of the AV. Importantly, we have demonstrated that women exhibit aberrant ECM remodeling, driven by enhanced MMP-1 and MMP-9. As well as lower TIMP-2 expressions. MMP-1 is one of the main enzymes responsible for the degradation of type I collagen and may play a role in regulating osteoblastic differentiation (31). MMP-related matrix degradation may also enhance the fragmentation of the elastic fibers (32) observed in histological preparations of women-derived AVs. Loss of elastic fibers jeopardizes the flexibility and stretch properties of the AV (33). The initiation of calcification requires a dynamic ECM remodeling. It has been described that MMP-1 knockdown resulted in enhanced expression of osteoblastic markers (31). MMP-9 also facilitates collagen remodeling. Expression of MMP-1 has been correlated with MMP-9 expression in AS patients, but not with MMP-3 (34). Moreover, the reduction in TIMP-2 found in AVs from women could enhance the collagenolytic activity of MMP-2 contributing to collagen type I reduction. On the basis of our findings, it is plausible that MMP-1 up-regulation found in women, could modulate collagen integrity and decrease osteoblastic differentiation, eventually delaying calcification.

Other factors might contribute to the prolonged ECM fibro-degrading phenotype in women and the late onset of calcific phenotypes. Sex-related transcriptomic signatures in cultured porcine VICs suggested that pro-fibrotic, pro-apoptotic and pro-calcific phenotype might be enhanced in male-derived VICs (6). In line with these results, enhanced apoptosis in men-derived AVs and VICs could be a source of apoptotic bodies that would enhance calcific phenotypes in men. Conversely, reduced apoptotic signals have been previously described in women diagnosed with rheumatic heart disease as compared to men, which could explain the decrease in mineralization (35). Moreover, it has been suggested that apoptotic and necrotic cell loss in heavily calcified AVs may lead to the development of prevalent dystrophic mineralization found in 83% of the histological AV specimens (36).

Our results are in accordance with other studies demonstrating that women, for similar AS severity, exhibit lower calcification than men (9, 11, 37). Nonetheless, a lower expression of well-known bone-related proteins explains the lower presence of calcification and mineralized bone in women-derived AVs (38–42). We confirmed these findings *in vitro*, demonstrating that the expression of osteogenic markers was constitutively lower in women. Either way, fibro-calcific process in men may elicit different mechanobiological and cellular responses resulting in different AS phenotypes relevant to the clinical outcome (33, 43). Altogether, our results discourage the use of sex-mixed VIC cultures and suggest that sex should be considered a potential experimental variable to bear in mind when developing new therapeutic approaches.

In conclusion, AS pathophysiology is different in women and men. AVs from women presented less inflammation, oxidative stress, calcification and apoptosis and enhanced ECM remodeling. Of special interest, AVs from women exhibited higher collagen type III content accompanied by increased MMP-1 and decreased TIMP-2 expression. Our study provides new molecular and cellular insights for the development of sex-specific strategies targeting differential pathophysiological processes contributing to AS.

## Limitations

This study had several limitations. First, our molecular approach may lead to methodological discrepancies with previous publications rather focused on histomorphometric or transcriptomic analyses. Our study specifically quantified collagen type I and III. Other collagens (not studied here) might contribute to histomorphometric differences. Second, the influence of other cell types presented in the AV (valve endothelial cells, inflammatory cells among others) has not been studied.

## Data Availability Statement

The original contributions presented in the study are included in the article/Supplementary Material, further inquiries can be directed to the corresponding author/s.

## Ethics Statement

The studies involving human participants were reviewed and approved by Comité Ético de Experimentación Clínica. Gobierno de Navarra, Departamento de Salud; Ethics numbers 17/2013 and PI2019/59. The patients/participants provided their written informed consent to participate in this study.

## Author Contributions

NL-A and EJ conceived and designed the study. LM, MG, VAr, AG-P, AF-C, AN, AG, VAl, and RS performed the data. LM, MG, VAr, AG-P, AF-C, AN, AG, VAl, RS, EJ, and NL-A analyzed and interpreted the data. LM, EJ, and NL-A led the design and drafted the article. All authors contributed to the article and approved the submitted version.

## Funding

This work was supported by Miguel Servet contract CP13/00221 from the Instituto de Salud Carlos III-FEDER, Fondo de Investigaciones Sanitarias [PI18/01875]. LM was supported by a PFIS (FI19/00302) grant, MG was supported by a Miguel Servet Foundation Ph.D. studentship, and EJ (CD19/00251) was supported by a Sara Borrell grant.

## Conflict of Interest

The authors declare that the research was conducted in the absence of any commercial or financial relationships that could be construed as a potential conflict of interest.

## Publisher's Note

All claims expressed in this article are solely those of the authors and do not necessarily represent those of their affiliated organizations, or those of the publisher, the editors and the reviewers. Any product that may be evaluated in this article, or claim that may be made by its manufacturer, is not guaranteed or endorsed by the publisher.

## Abbreviations

AS: Aortic stenosis
AU: Arbitrary Units
AV: Aortic valve
Bax: BCL-2 associated protein
BMP: Bone morphogenetic protein
BNP: Brain natriuretic peptide
CCL2: C-C motif chemokine ligand 2
ECM: Extracellular matrix
eNOS: endothelial Nitric Oxide synthase
FBS: Fetal bovine serum
FGF: Fibroblast growth factor
IL: interleukin
Lox-1: Lysil oxidase-1
MMP: Matrix metalloproteinase
PARP1: Poly [ADP-ribose] polymerase 1
Rank-L: Receptor activator of NF-kappa-B ligand
Runx2: Runt-related transcription factor 2
Sox-9: SRY-box transcription factor 9
TGF-β: Tissue growth factor-beta
TIMP: Tissue inhibitor metalloproteinase
VIC: Valve interstitial cell
WB: Western blot
α-SMA: α smooth muscle actin.

## References

[1] Goody P Hosen M Christmann D Niepmann S Zietzer A Adam M. Aortic valve stenosis: from basic mechanisms to novel therapeutic targets. Arterioscler Thromb Vasc Biol. (2020) 40:885–900. 10.1161/ATVBAHA.119.313067 32160774

[2] Nishimura R Otto C Bonow R Carabello B Erwin J Guyton R et al. 2014 AHA/ACC Guideline for the Management of Patients With Valvular Heart Disease: a report of the American College of Cardiology/American Heart Association Task Force on Practice Guidelines. Circulation. (2014) 129:e521–643. 10.1161/CIR.0000000000000031 24589853

[3] Yadgir S Johnson C Aboyans V Adebayo O Adedoyin R Afarideh M. Global, regional, and national burden of calcific aortic valve and degenerative mitral valve diseases, 1990-2017. Circulation. (2020) 141:1670–80. 10.1161/CIR.0000000000000848 32223336

[4] Otto CM Kuusisto J Reichenbach DD Gown AM O'brien KD. Characterization of the early lesion of “degenerative” valvular aortic stenosis histological and immunohistochemical studies. Circulation. (1994) 90:844–53. 10.1161/01.CIR.90.2.844 7519131

[5] Rutkovskiy A Malashicheva A Sullivan G Bogdanova M Kostareva A Stensløkken K et al. Valve interstitial cells: the key to understanding the pathophysiology of heart valve calcification. J Am Heart Assoc. (2017) 6:e006339. 10.1161/JAHA.117.006339 28912209 PMC5634284

[6] McCoy C Nicholas D Masters K. Sex-related differences in gene expression by porcine aortic valvular interstitial cells. PLoS ONE. (2012) 7:e39980. 10.1371/journal.pone.0039980 22808080 PMC3393722

[7] Boskovski MT Gleason TG. Current therapeutic options in aortic stenosis. Circ Res. (2021) 128:1398–417. 10.1161/CIRCRESAHA.121.318040 33914604

[8] Summerhill V Moschetta D Orekhov A Poggio P Myasoedova V. Sex-specific features of calcific aortic valve disease. Int J Mol Sci. (2020) 21:1–19. 10.3390/ijms21165620 32781508 PMC7460640

[9] Simard L Côté N Dagenais F Mathieu P Couture C Trahan S et al. Sex-related discordance between aortic valve calcification and hemodynamic severity of aortic stenosis: is valvular fibrosis the explanation? Circ Res. (2017) 120:681–91. 10.1161/CIRCRESAHA.116.309306 27879282

[10] Zhang B Miller V Miller J. Influences of sex and estrogen in arterial and valvular calcification. Front Endocrinol. (2019) 10:622. 10.3389/fendo.2019.00622 31620082 PMC6763561

[11] Voisine M Hervault M Shen M Boilard A Filion B Rosa M. Age, sex, and valve phenotype differences in fibro-calcific remodeling of calcified aortic valve. J Am Heart Assoc. (2020) 9:e015610. 10.1161/JAHA.119.015610 32384012 PMC7660864

[12] Neumann F Sousa-Uva M Ahlsson A Alfonso F Banning A Benedetto U. 2018 ESC/EACTS Guidelines on myocardial revascularization. Eur Heart J. (2019) 40:87–165. 10.1093/eurheartj/ehy855 30165437

[13] Sádaba J Martínez-Martínez E Arrieta V Álvarez V Fernández-Celis A Ibarrola J. Role for galectin-3 in calcific aortic valve stenosis. J Am Heart Assoc. (2016) 5:e004360. 10.1161/JAHA.116.004360 27815266 PMC5210369

[14] Ibarrola J Matilla L Martínez-Martínez E Gueret A Fernández-Celis A Henry J et al. Myocardial injury after ischemia/reperfusion is attenuated by pharmacological galectin-3 inhibition. Sci Rep. (2019) 9:9607. 10.1038/s41598-019-46119-6 31270370 PMC6610618

[15] Masjedi S Ferdous Z. Understanding the role of sex in heart valve and major vascular diseases. Cardiovasc Eng Technol. (2015) 6:209–19. 10.1007/s13239-015-0226-x 26577355

[16] Mahmoudi M Gormaz J Erazo M Howard M Baeza C Feelisch M et al. Early oxidative stress response in patients with severe aortic stenosis undergoing transcatheter and surgical aortic valve replacement: a transatlantic study. Oxid Med Cell Longev. (2019) 2019:6217837. 10.1155/2019/6217837 31827686 PMC6881568

[17] Marincheva-Savcheva G Subramanian S Qadir S Figueroa A Truong Q Vijayakumar J et al. Imaging of the aortic valve using fluorodeoxyglucose positron emission tomography increased valvular fluorodeoxyglucose uptake in aortic stenosis. J Am Coll Cardiol. (2011) 57:2507–15. 10.1016/j.jacc.2010.12.046 21679852

[18] New S Aikawa E. Molecular imaging insights into early inflammatory stages of arterial and aortic valve calcification. Circ Res. (2011) 108:1381–91. 10.1161/CIRCRESAHA.110.234146 21617135 PMC3139950

[19] Dweck MR Boon NA Newby DE. Calcific aortic stenosis a disease of the valve and the myocardium. J Am Coll Cardiol. (2012) 60:1854–63. 10.1016/j.jacc.2012.02.093 23062541

[20] Miller J Chu Y Brooks R Richenbacher W Peña-Silva R Heistad D. Dysregulation of antioxidant mechanisms contributes to increased oxidative stress in calcific aortic valvular stenosis in humans. J Am Coll Cardiol. (2008) 52:843–50. 10.1016/j.jacc.2008.05.043 18755348 PMC2748760

[21] Parra-Izquierdo I Castaños-Mollor I López J Gómez C San Román J Sánchez Crespo M et al. Calcification induced by type i interferon in human aortic valve interstitial cells is larger in males and blunted by a janus kinase inhibitor. Arterioscler Thromb Vasc Biol. (2018) 38:2148–59. 10.1161/ATVBAHA.118.311504 30026273

[22] Odelin G Faure E Maurel-Zaffran C Zaffran S. Krox20 regulates endothelial nitric oxide signaling in aortic valve development and disease. J Cardiovasc Dev Dis. (2019) 6:39. 10.3390/jcdd6040039 31684048 PMC6955692

[23] Parra-Izquierdo I Castaños-Mollor I López J Gómez C San Román JA Sánchez Crespo M et al. Lipopolysaccharide and interferon-γ team up to activate HIF-1α via STAT1 in normoxia and exhibit sex differences in human aortic valve interstitial cells. Biochim Biophys Acta Mol basis Dis. (2019) 1865:2168–79. 10.1016/j.bbadis.2019.04.014 31034990

[24] Sarajlic P Plunde O Franco-Cereceda A Bäck M. Artificial intelligence models reveal sex-specific gene expression in aortic valve calcification. JACC Basic to Transl Sci. (2021) 6:403–12. 10.1016/j.jacbts.2021.02.005 34095631 PMC8165113

[25] Fayet C Bendeck M Gotlieb A. Cardiac valve interstitial cells secrete fibronectin and form fibrillar adhesions in response to injury. Cardiovasc Pathol. (2007) 16:203–11. 10.1016/j.carpath.2007.02.008 17637428

[26] Combs M Yutzey K. Heart valve development: regulatory networks in development and disease. Circ Res. (2009) 105:408–21. 10.1161/CIRCRESAHA.109.201566 19713546 PMC2777683

[27] Chaterji S Lam C Ho D Proske D Baker A. Syndecan-1 regulates vascular smooth muscle cell phenotype. PLoS ONE. (2014) 9:e89824. 10.1371/journal.pone.0089824 24587062 PMC3934950

[28] Jover E Silvente A Marín F Martínez-González J Orriols M Martinez C et al. Inhibition of enzymes involved in collagen cross-linking reduces vascular smooth muscle cell calcification. FASEB J. (2018) 32:4459–69. 10.1096/fj.201700653R 29547702

[29] Martínez-Martínez E Rodríguez C Galán M Miana M Jurado-López R Bartolomé M et al. The lysyl oxidase inhibitor (β-aminopropionitrile) reduces leptin profibrotic effects and ameliorates cardiovascular remodeling in diet-induced obesity in rats. J Mol Cell Cardiol. (2016) 92:96–104. 10.1016/j.yjmcc.2016.01.012 26780438

[30] Syväranta S Alanne-Kinnunen M Oörni K Oksjoki R Kupari M Kovanen P. Potential pathological roles for oxidized low-density lipoprotein and scavenger receptors SR-AI, CD36, and LOX-1 in aortic valve stenosis. Atherosclerosis. (2014) 235:398–407. 10.1016/j.atherosclerosis.2014.05.933 24929820

[31] Hayami T Kapila Y Kapila S. MMP-1 (collagenase-1) and MMP-13 (collagenase-3) differentially regulate markers of osteoblastic differentiation in osteogenic cells. Matrix Biol. (2008) 27:682–92. 10.1016/j.matbio.2008.07.005 18755271 PMC2683744

[32] Di Vito A Donato A Presta I Mancuso T Brunetti F Mastroroberto P et al. Extracellular matrix in calcific aortic valve disease: architecture, dynamic and perspectives. Int J Mol Sci. (2021) 22:1–21. 10.3390/ijms22020913 33477599 PMC7831300

[33] Kodigepalli K Thatcher K West T Howsmon D Schoen F Sacks M. Biology and biomechanics of the heart valve extracellular matrix. J Cardiovasc Dev Dis. (2020) 7:1–22. 10.3390/jcdd7040057 33339213 PMC7765611

[34] Lurins J Lurina D Svirskis S Nora-Krukle Z Tretjakovs P Mackevics V et al. Impact of several proinflammatory and cell degradation factors in patients with aortic valve stenosis. Exp Ther Med. (2019) 17:2433–42. 10.3892/etm.2019.7254 30906430 PMC6425154

[35] Xiao F Zheng R Yang D Cao K Zhang S Wu B et al. Sex-dependent aortic valve pathology in patients with rheumatic heart disease. PLoS ONE. (2017) 12. 10.1371/journal.pone.0180230 28662157 PMC5491156

[36] Mohler III ER Gannon F Reynolds C Zimmerman R Keane MG Kaplan FS. Bone formation and inflammation in cardiac valves. Circulation. (2001) 103:1522–8. 10.1161/01.CIR.103.11.1522 11257079

[37] Aggarwal S Clavel M Messika-Zeitoun D Cueff C Malouf J Araoz P. Sex differences in aortic valve calcification measured by multidetector computed tomography in aortic stenosis. Circ Cardiovasc Imaging. (2013) 6:40–7. 10.1161/CIRCIMAGING.112.980052 23233744

[38] Chen L Jiang W Huang J He B Zuo G Zhang W. Insulin-like growth factor 2 (IGF-2) potentiates BMP-9-induced osteogenic differentiation and bone formation. J Bone Miner Res. (2010) 25:2447–59. 10.1002/jbmr.133 20499340 PMC3179288

[39] Pawade T Newby D Dweck M. Calcification in aortic stenosis: the skeleton key. J Am Coll Cardiol. (2015) 66:561–77. 10.1016/j.jacc.2015.05.066 26227196

[40] Lerman D Prasad S Alotti N. Calcific aortic valve disease: molecular mechanisms and therapeutic approaches. Eur Cardiol. (2015) 10:108–12. 10.15420/ecr.2015.10.2.108 27274771 PMC4888946

[41] O'Brien KD Kuusisto J Reichenbach DD Ferguson M Giachelli C Alpers CE et al. Osteopontin is expressed in human aortic valvular lesions. Circulation. (1995) 92:2163–68. 10.1161/01.CIR.92.8.2163 7554197

[42] Peacock J Levay A Gillaspie D Tao G Lincoln J. Reduced sox9 function promotes heart valve calcification phenotypes *in vivo*. Circ Res. (2010) 106:712–9. 10.1161/CIRCRESAHA.109.213702 20056916 PMC2863131

[43] Yip C Chen J Zhao R Simmons C. Calcification by valve interstitial cells is regulated by the stiffness of the extracellular matrix. Arterioscler Thromb Vasc Biol. (2009) 29:936–42. 10.1161/ATVBAHA.108.182394 19304575

