# Supplementary material for: Sex-Differences in Aortic Stenosis: Mechanistic Insights and Clinical Implications
Source: Front Cardiovasc Med. 2022 Feb 24;9:818371. doi: 10.3389/fcvm.2022.818371 (PMC8907577; doi:10.3389/fcvm.2022.818371)
Supplement: Supplementary file 1 [file Data_Sheet_1.pdf]

Supplemental table I. Demographic & clinical data of VICs donors.

	Total	Men	Women	p-value
n (%)	36 (100)	24 (66.67)	12 (33.33)	
Age (median [IQR])	69.5 [65-77.8]	69 [65-75.80]	74.5 [66.50-79.00]	0.233
Weight (mean ± SD)	77.5 ± 13.0	82.30 ± 10.5	67.80 ± 12.40	0.0008
Height (mean ± SD)	164 ± 9.36	168 ± 6.61	155 ± 7.23	< 0.0001
Body surface (mean ± SD)	1.79 ± 0.208	1.86 ± 0.203	1.66 ± 0.153	0.0057
DM, n (%)	1 (2.78)	1 (4.17)	0	0.473
HTA, n (%)	20 (55.55)	16 (66.67)	4 (33.33)	0.058
**Drug medicines**				
ACEI, n (%)	7 (19.44)	5 (20.83)	2 (16.67)	0.766
ARB, n (%)	9 (25)	7 (29.17)	2 (16.67)	0.414
Spironolactone, n (%)	1 (2.78)	0	1 (8.33)	0.151
Eplerenona, n (%)	0	0	0	.
Diuretics, n (%)	15 (41.67)	9 (37.50)	6 (50)	0.473
B-blockers, n (%)	10 (27.78)	6 (25)	4 (33.33)	0.599
Statins, n (%)	19 (52.78)	13 (54.17)	6 (50)	0.813
**Biochemical analyses**				
Total cholesterol, mg/dL (mean ± SD)	177 ± 44.20	174 ± 46.20	184 ± 41	0.553
Triglycerides, mg/dL (median [IQR])	93.5 [62.30-111]	96.5 [68.30-111]	89 [60.3-110]	0.54
HDL, mg/dL (mean ± SD)	45.8 ± 10.70	43.40 ± 10	50.70 ± 10.80	0.054
LDL, mg/dL (mean ± SD)	110 ± 35	108 ± 35.50	115 ± 35.10	0.618
**Echocardiographic parameters**				
Max gradient (median [IQR])	75.50 [70-85.80]	75 [70.30-79.80]	80 [64-111]	0.456
Medium gradient (median [IQR])	49.5 [44.30-54.80]	49 [45.30-52]	50.50 [40.80-68.50]	0.585
Valvular Area echocardiography, cm2 (median [IQR])	0.70 [0.60-0.80]	0.8 [0.6-0.8]	0.60 [0.46-0.87]	0.272
EF % (median [IQR])	65 [60-73]	65 [57-70]	66 [60-75]	0.637

N, sample size (biological replicates); SD, standard deviation; DM, diabetes mellitus; HTA, arterial hypertension; BAV, bicuspid aortic valve; ACEI, angiotensin converting enzyme inhibitors; ARB, angiotensin II receptor blockers; HDL, high density lipoproteins; LDL, low density lipoprotein; EF, ejection fraction.

Supplemental table II. Multivariate models of variables that showed independent association with sex after adjustment for age, statin use and Total cholesterol.

Treatments

Diuretics		Estimate	Pr(>|z|)
(Intercept)		-2.421242	0.1270
Sex	*male*	*Reference*	
	female	0.772818	0.0288 *
Age		0.045367	0.0147 *
Statin treatment	*No*	*Reference*	
	Yes	0.249459	0.4878
Total Cholesterol		-0.005693	0.1844

Cardiovalvular characterization

Valve area MRI (cm^2)		Estimate	Pr(>|z|)
(Intercept)		0.9283941	1.58e-06 ***
Sex	*male*		*Reference*
	female	-0.0815313	0.0308 *
Age		-0.0001477	0.9391
Statin treatment	*No*		*Reference*
	Yes	0.0352889	0.3786
Total Cholesterol		-0.0005032	0.3217

Laboratory variables

Body surface (cm^2)		Estimate	Pr(>|z|)
(Intercept)		2.0685887	< 2e-16 ***
Sex	*male*	*Reference*	
	female	-0.2150957	3.87e-16 ***
Age		-0.0018695	0.1275
Statin treatment	*No*	*Reference*	
	Yes	0.0546841	0.0292 *
Total Cholesterol		-0.0004039	0.1745

Demographic variables

Height (cm)		Estimate	Pr(>|z|)
(Intercept)		181.21604	< 2e-16 ***
Sex	*male*	*Reference*	
	female	-12.45500	< 2e-16 ***
Age		-0.16739	0.000396 ***
Statin treatment	*No*	*Reference*	
	Yes	-0.01456	0.987727
Total Cholesterol		-0.01146	0.309903

Weight (Kg)		Estimate	Pr(>|z|)
(Intercept)		87.11759	< 2e-16 ***
Sex	*male*		*Reference*
	female	-11.13625	1.54e-07 ***
Age		-0.06629	0.5227
Statin treatment	*No*		*Reference*
	Yes	5.43340	0.0107 *
Total Cholesterol		-0.02711	0.2817

ELISA targets

IL-6		Estimate	Pr(>|z|)
(Intercept)		23.35415	0.69772
Sex	male	Reference	
	female	-33.0810	0.00751 **
Age		0.41013	0.49952
Statin treatment	No	Reference	
	Yes	-27.00411	0.05748
Total Cholesterol		0.02605	0.88367

Syndecan-1		Estimate	Pr(>|z|)
(Intercept)		-19.3702	0.9703
Sex	male		Reference
	female	-234.4514	0.0387 *
Age		10.0458	0.0718 .
Statin treatment	No		Reference
	Yes	14.5504	0.9069
Total Cholesterol		-0.3158	0.8368

RANTES		Estimate	Pr(>|z|)
(Intercept)		11.8755651	0.34078
Sex	*male*	*Reference*	
	female	-8.7312512	0.00119 **
Age		-0.0008782	0.99478
Statin treatment	*No*	*Reference*	
	Yes	0.1192175	0.96677
Total Cholesterol		0.0535902	0.13243

MMP-1		Estimate	Pr(>|z|)
(Intercept)		355.654	0.342171
Sex	*male*		*Reference*
	female	299.646	0.000226 ***
Age		-1.620	0.689757
Statin treatment	*No*		*Reference*
	Yes	65.065	0.452831
Total Cholesterol		-1.058	0.316660

eNOS		Estimate	Pr(>|z|)
(Intercept)		-260.6195	0.280097
Sex	*male*	*Reference*	
	female	-176.4272	0.000394 ***
Age		6.8979	0.010329 *
Statin treatment	*No*	*Reference*	
	Yes	-56.3297	0.277449
Total Cholesterol		0.3322	0.599379

MMP-9		Estimate	Pr(>|z|)
(Intercept)		132.01989	0.35135
Sex	*male*		*Reference*
	female	84.58472	0.00311 **
Age		-0.59876	0.66486
Statin treatment	*No*		*Reference*
	Yes	0.18719	0.99501
Total Cholesterol		-0.03238	0.93949

Osteopontin		Estimate	Pr(>|z|)
(Intercept)		723.833	0.4342
Sex	*male*	*Reference*	
	female	-439.170	0.0284 *
Age		6.703	0.5056
Statin treatment	*No*	*Reference*	
	Yes	66.337	0.7584
Total Cholesterol		2.828	0.2694

TIMP-2		Estimate	Pr(>|z|)
(Intercept)		632.302	0.0729 .
Sex	*male*		*Reference*
	female	-164.340	0.0235 *
Age		-4.717	0.1968
Statin treatment	*No*		*Reference*
	Yes	7.577	0.9255
Total Cholesterol		1.930	0.0604 .

BMP-9		Estimate	Pr(>|z|)
(Intercept)		129.941412	0.0193 *
Sex	*male*	*Reference*	
	female	-19.836459	0.0631 .
Age		-1.016870	0.0931 .
Statin treatment	*No*	*Reference*	
	Yes	13.621559	0.2386
Total Cholesterol		-0.005864	0.9672

Osteocalcin		Estimate	Pr(>|z|)
(Intercept)		411.4616	0.3423
Sex	*male*		*Reference*
	female	-171.6132	0.0593
Age		-0.0648	0.9888
Statin treatment	*No*		*Reference*
	Yes	47.1773	0.6350
Total Cholesterol		0.8531	0.4840

RANKL		Estimate	Pr(>|z|)
(Intercept)		953.2434	0.1242
Sex	*male*	*Reference*	
	female	196.6640	0.0908 .
Age		1.1003	0.8690
Statin treatment	*No*	*Reference*	
	Yes	56.6361	0.6715

Total Cholesterol		0.2662	0.8749

Western blot/zymography targets

MMP-1 zymography		Estimate	Pr(>|z|)
(Intercept)		3.2028646	0.0027 **
Sex	*male*	*Reference*	
	female	0.5094750	0.0240 *
Age		-0.0228582	0.0400 *
Statin treatment	*No*	*Reference*	
	Yes	-0.2616957	0.2773
Total Cholesterol		-0.0008033	0.7937

BAX		Estimate	Pr(>|z|)
(Intercept)		0.629794	0.5202
Sex	*male*		*Reference*
	female	-0.486511	0.0199 *
Age		0.016152	0.1187
Statin treatment	*No*		*Reference*
	Yes	-0.208304	0.3168
Total Cholesterol		-0.003153	0.2826

Sox9		Estimate	Pr(>|z|)
(Intercept)		2.250635	0.07633
Sex	*male*	*Reference*	
	female	-0.699108	0.00603 **
Age		0.012132	0.33333
Statin treatment	*No*	*Reference*	
	Yes	-0.552007	0.05182
Total Cholesterol		-0.007945	0.04069 *

Collagen-3		Estimate	Pr(>|z|)
(Intercept)		0.999518	0.4392
Sex	*male*		*Reference*
	female	0.659427	0.0244 *
Age		0.005519	0.6859
Statin treatment	*No*		*Reference*
	Yes	-0.152407	0.6325
Total Cholesterol		-0.002518	0.5324

cd68		Estimate	Pr(>|z|)
(Intercept)		-0.0144286	0.9359
Sex	*male*	*Reference*	
	female	-0.0982707	0.0106 *
Age		0.0020559	0.2837
Statin treatment	*No*	*Reference*	
	Yes	-0.0542416	0.1822
Total Cholesterol		0.0004078	0.4309

TGF-β		Estimate	Pr(>|z|)
(Intercept)		3.142944	0.3196
Sex	*male*		*Reference*
	female	-1.234233	0.0473 *
Age		0.013526	0.6906
Statin treatment	*No*		*Reference*
	Yes	-0.406679	0.5324
Total Cholesterol		-0.005277	0.5253

CD45		Estimate	Pr(>|z|)
(Intercept)		1.125e-01	0.5394
Sex	*male*	*Reference*	
	female	-1.015e-01	0.0125 *
Age		1.933e-03	0.2912
Statin treatment	*No*	*Reference*	
	Yes	-3.198e-02	0.4855
Total Cholesterol		3.677e-05	0.9496

Fibronectin		Estimate	Pr(>|z|)
(Intercept)		-260.393	0.54588
Sex	*male*		*Reference*
	female	-23.6892	0.00244 **
Age		0.5749	0.21128
Statin treatment	*No*		*Reference*
	Yes	-7.9581	0.35475
Total Cholesterol		0.1057	0.38504

Nitrotyrosine		Estimate	Pr(>|z|)
(Intercept)		0.100703	0.86963

Sex	*male*	*Reference*	
	female	-0.394294	0.00195 *
Age		0.011426	0.11127
Statin treatment	*No*	*Reference*	
	Yes	0.101977	0.45917
Total Cholesterol		-0.001876	0.27641

RT-PCR targets (mRNA)

RUNX2		Estimate	Pr(>|z|)	*COL3A1*		Estimate	Pr(>|z|)
(Intercept)		1.098189	0.2171	(Intercept)		1.0587677	0.2824
Sex	*male*	*Reference*		Sex	*male*		*Reference*
	female	-0.474281	0.0283 *		*female*	0.4534495	0.0361 *
Age		0.008967	0.3593	Age		-0.0005736	0.9547
Statin treatment	*No*	*Reference*		Statin treatment	*No*		*Reference*
	Yes	-0.384092	0.0886 .		*Yes*	-0.0533679	0.8162
Total Cholesterol		-0.003360	0.2106	Total Cholesterol		0.0010768	0.7163

Full unedited gel for Figure 1D and 1H

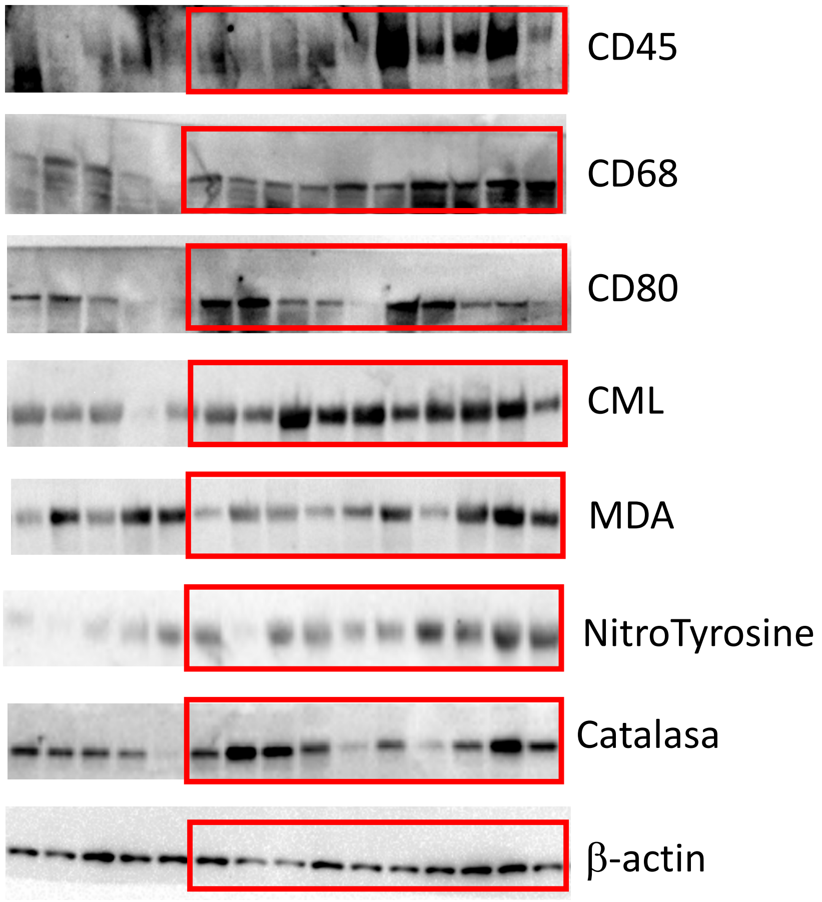

Full unedited gel for Figure 1H

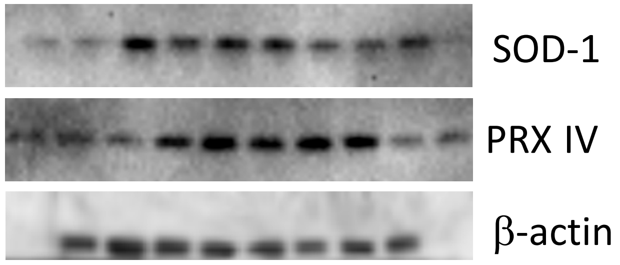

Full unedited gel for Figure 2D, 3D and 3G

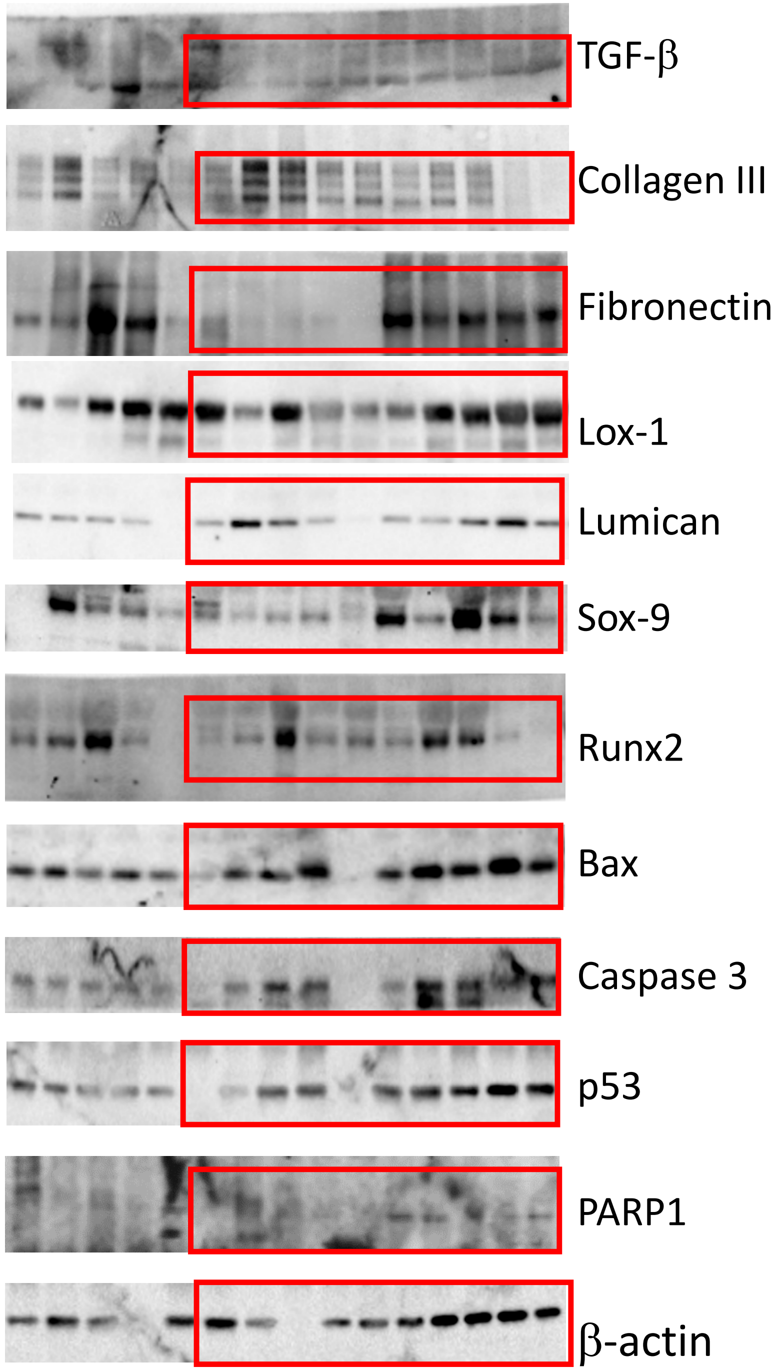

Full unedited zymography for Supplemental Figure IIIE and for Figure 4I

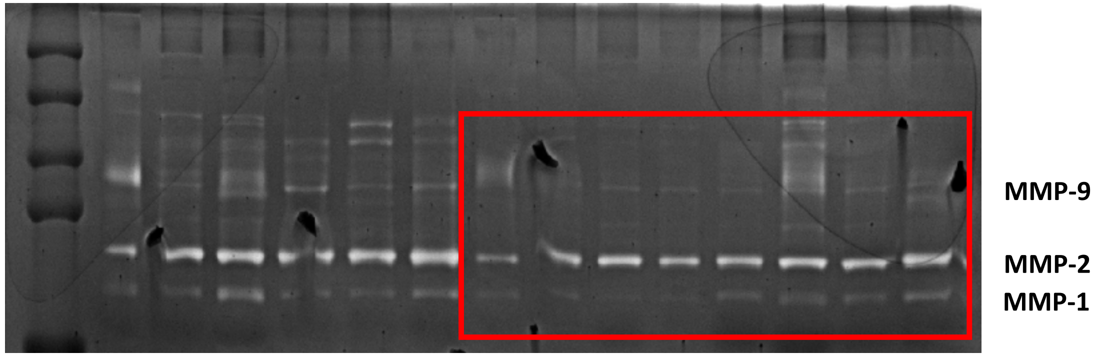

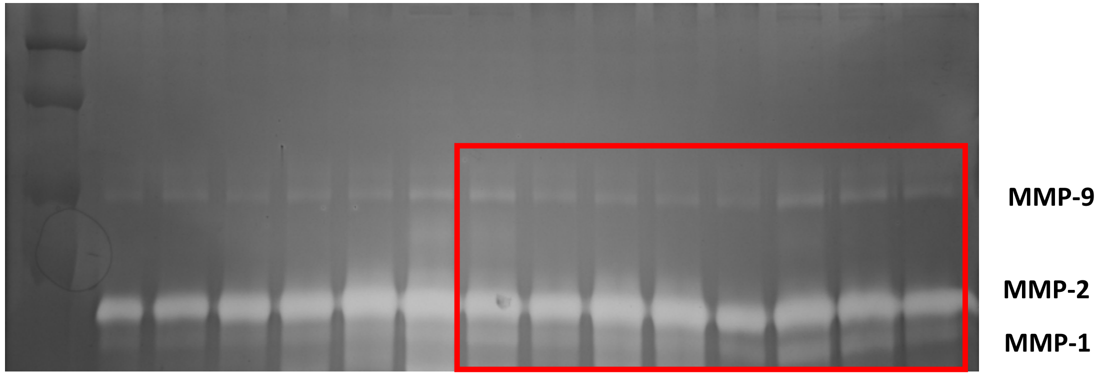

Full unedited gel for Figure 4N

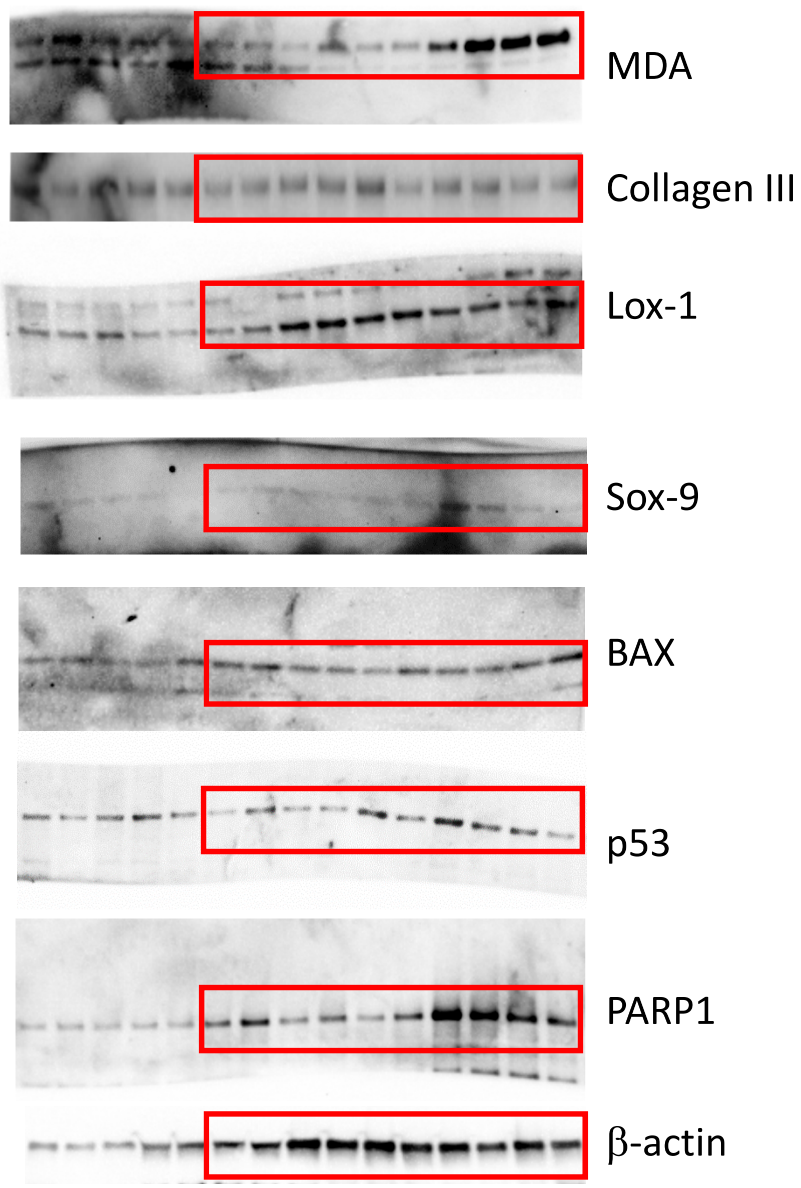

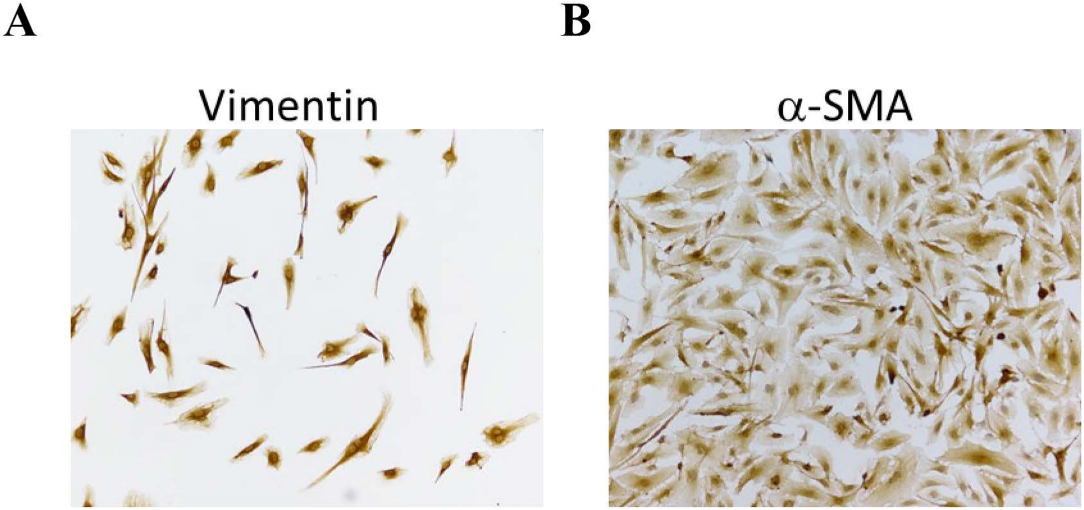

Supplemental figure I

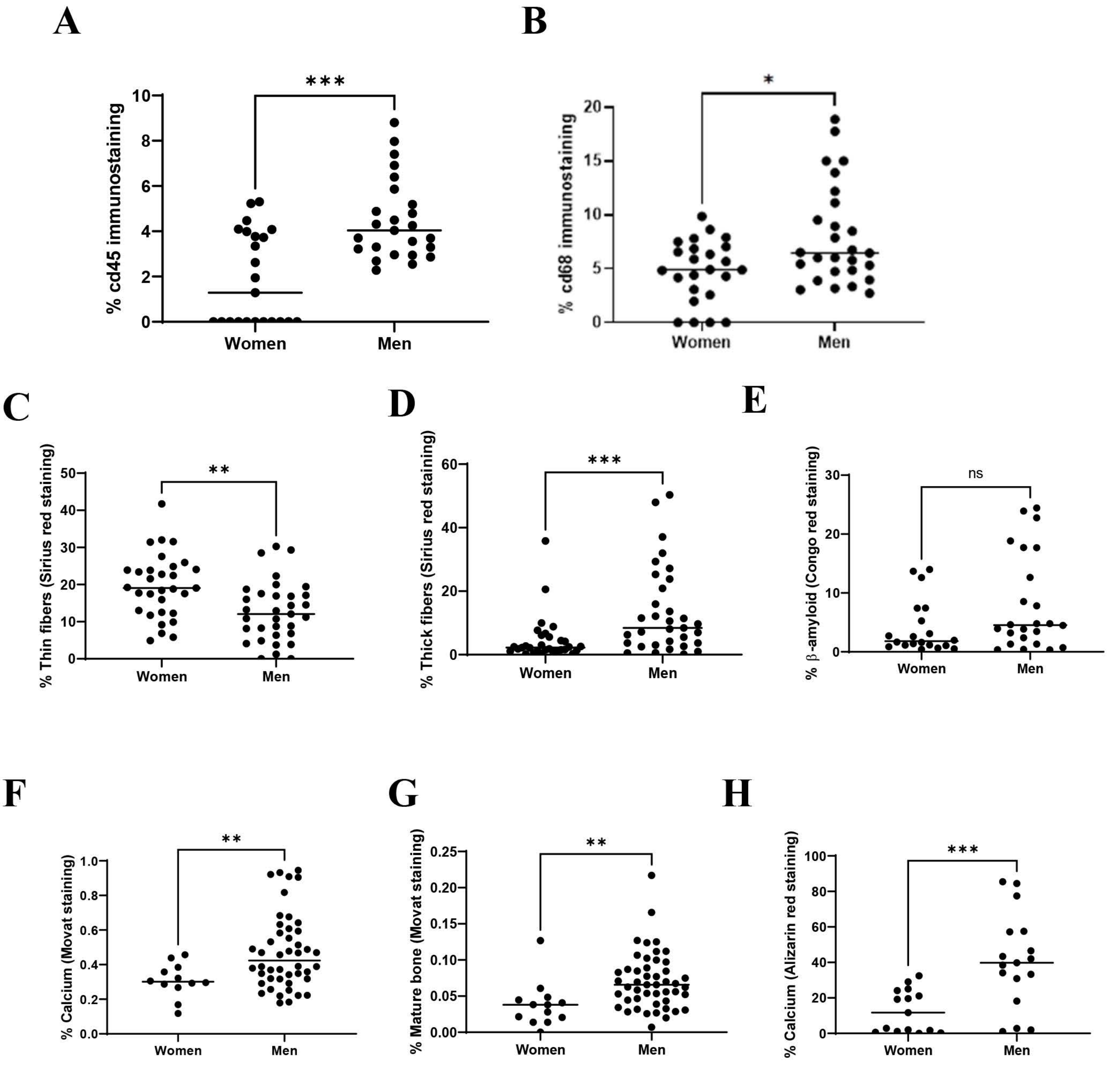

Supplemental figure II

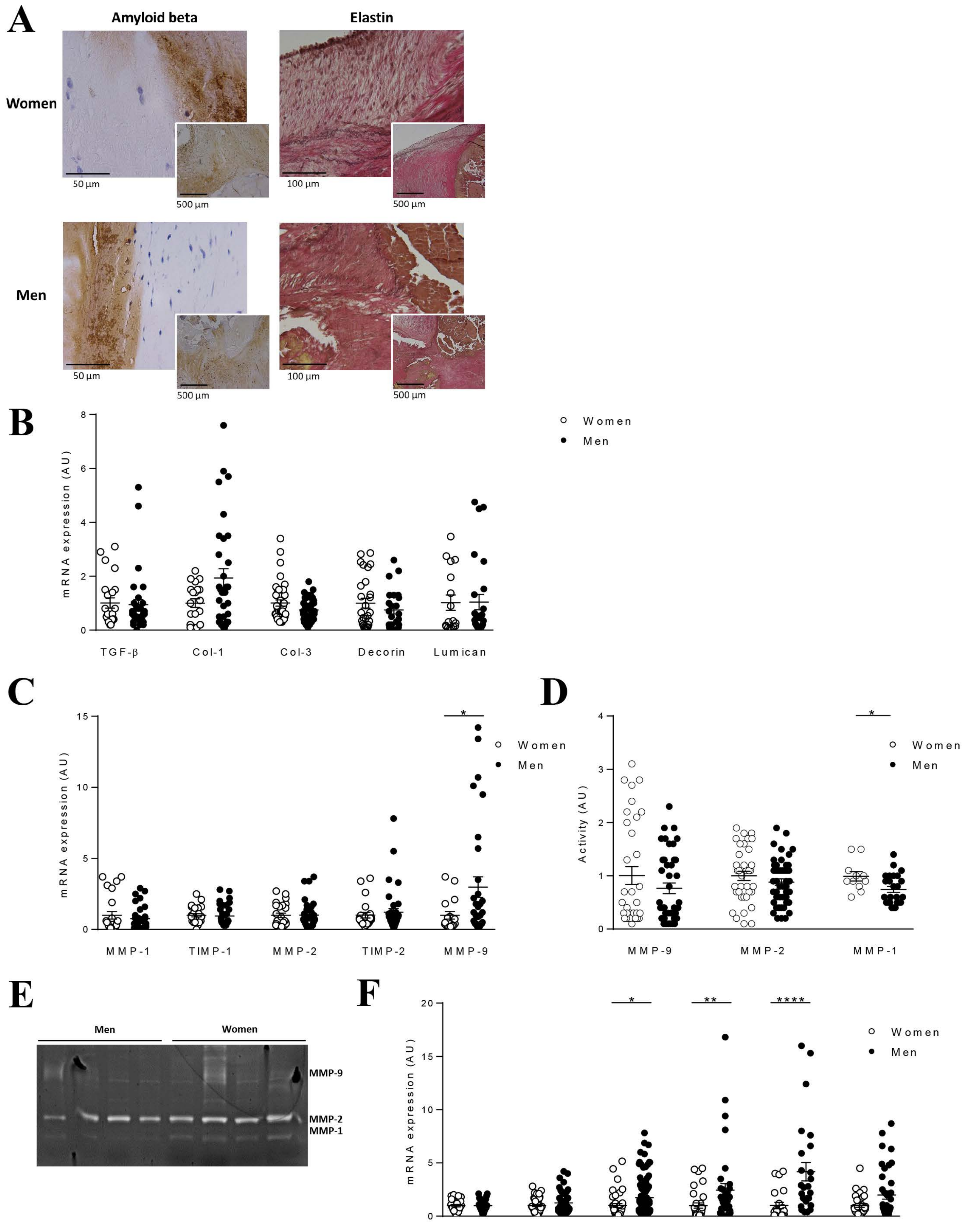

Supplemental figure III

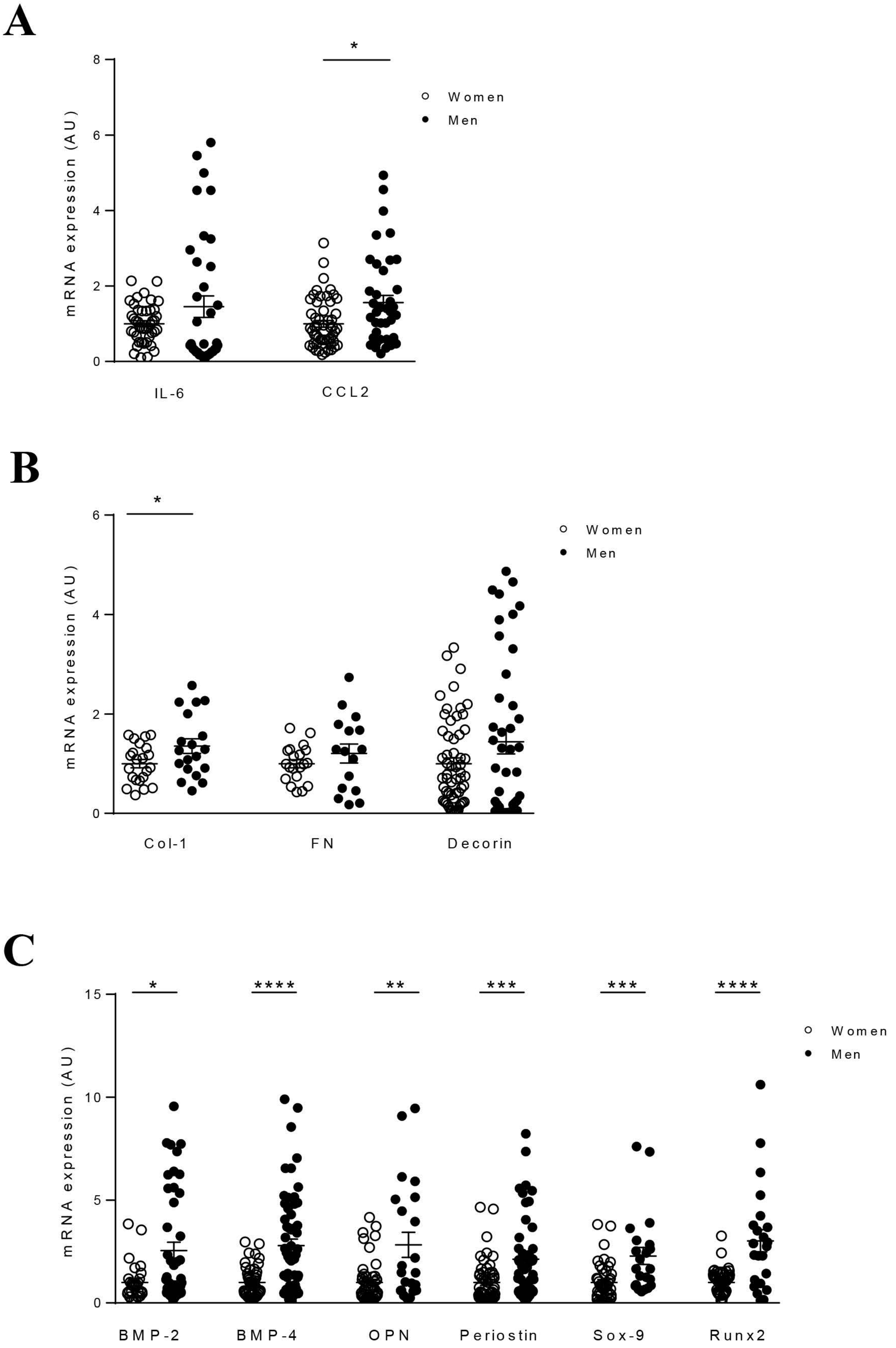

Supplemental figure IV

SUPPLEMENTAL FIGURE LEGENDS

Supplemental Figure I. Phenotype characterization of VICs isolated from human explanted AVs by immunocytochemistry. VICs isolated from women and men patients undergoing elective surgical AV replacement were routinely assessed for antigenic characterization by vimentin (A) and alpha-smooth muscle actin (α-SMA) (B) immunocytochemistry.

Supplemental Figure II. Digital image analyses plots of histological preparations. Human AV preparations were immunohistochemically and histologically assessed in multiple biological replicates *per* sex and quantified using digital image analyses. CD68 immunohistochemistry (A) was quantified in AV preparations from 24 women and 27 men. CD45 immunohistochemistry (B) was quantified in AV preparations from 22 women and 26 men. Thin (C) and thick (D) collagen fibers were quantified in polarized images of AV preparations from 31 women and 33 men stained with Sirius red die. Beta-amyloid deposits (E) were quantified in polarized images of AV preparations from 20 women and 24 men stained with Congo red die. Calcium deposits (F) (bright red areas) and bone presence (G) (bright yellow areas) were further quantified in AV preparations from 12 women and 46 men stained using Movat pentachrome method. Calcium deposits (H) were validated in AV preparations from 15 women and 18 men stained with Alizarin Red. *$p<0.05$ vs Women.

Supplemental Figure III. Sex differences in inflammation and oxidative stress markers in AVs from AS patients. Representative images of β-amyloid immunostaining and elastin staining in women and men AVs (A). mRNA expression of fibrosis markers (B) and

ECM remodeling molecules (C). Activity of metalloproteinases (D) and representative zymograme of MMP-9, -2 and -1 (E). Gene expression of calcification markers (F). AV: aortic valve; AS: aortic stenosis; ECM: extracellular matrix; TGF-β: transforming Growth Factor-β1; Col: collagen; MMP: matrix metalloproteinase; TIMP: metalloproteinase inhibitor; BMP: bone morphogenetic protein; OPN: osteopontin; Sox-9: SRY (sex-determining region Y)-box 9; Runx2: runt-related transcription factor 2. Gene expression data were normalized to HPRT, β-actin and GADPH. Dot plots represent mean and standard error of the mean (SEM) of each group of subjects in arbitrary units (AU). All data were normalized to women. *$p<0.05$ vs Women.

Supplemental Figure IV. Sex differences in mRNA expression of human VICs. mRNA expression of inflammatory (A), fibrosis (B) and calcification markers (C). IL: interleukin; CCL2: C-C Motif Chemokine Ligand 2; Col-1: collagen type 1; FN: fibronectin; BMP: bone morphogenetic protein; OPN: osteopontin; Sox-9: SRY (sex-determining region Y)-box 9; Runx2: runt-related transcription factor 2. Gene expression data were normalized to HPRT, β-actin and GADPH. Dot plots represent mean and standard error of the mean (SEM) of each group of subjects (Women n=12 and men n=24, VICs from each patient have 3-8 replicates) in arbitrary units (AU). All data were normalized to women. *$p<0.05$ vs Women.

Major Resources Table

In order to allow validation and replication of experiments, all essential research materials listed in the Methods should be included in the Major Resources Table below. Authors are encouraged to use public repositories for protocols, data, code, and other materials and provide persistent identifiers and/or links to repositories when available. Authors may add or delete rows as needed.

Antibodies

Target antigen	Vendor or Source	Catalog #	Working concentration
Vimentin	Santa Cruz	sc-373717	2 µg/ml
Lox-1	Novus Biological	NB100-2527	13 µg/ml
Sox-9	Millipore	AB5535	10 µg/ml
MDA	Abcam	ab6463	8 µg/ml
Bax	Santa Cruz	Sc-493	1 µg/ml
Caspase-3	Cell Signalling	CST#9662	0.49 µg/ml
p53	Santa Cruz	Sc-55476	2 µg/ml
Col-3	Santa Cruz	Sc-28888	4 µg/ml
PARP-1	Cell Signalling	CST#9542	0.976 µg/ml
CD45	Santa Cruz	Sc-1178	2 µg/ml
CD68	Santa Cruz	Sc-17832	2 µg/ml
CD80	Santa Cruz	sc-376012	2 µg/ml
CML	Abcam	Ab27684	6 µg/ml
Nitrotyrosine	Santa Cruz	sc-32757	2 µg/ml
SOD-1	Cell Signalling	CST#4266	1 µg/ml
PRXIV	Santa Cruz	Sc-376668	2 µg/ml
Catalase	Santa Cruz	Sc-271803	2 µg/ml
Runx2	Santa Cruz	sc-101145	1 µg/ml
Lumican	Abcam	ab108286	10,03 µg/ml
FN	Millipore	MAB1926	10 µg/ml
TGF-β	Santa Cruz	Sc-130348	1 µg/ml
β-Actin	Sigma	A5441	2.5 µg/ml
Anti-mouse	Sigma Aldrich	GENA931-1ML	
Anti-rabbit	Sigma Aldrich	GENA934-1ML	

ELISA

Target antigen	Vendor or Source	Catalog #
IL-6	R&D Systems	DY206
CCL2	R&D Systems	DY279
Rantes	R&D Systems	DY278
Col-1	R&D Systems	DY6220-05
FN	R&D Systems	DY1918
Decorin	R&D Systems	DY143
Lumican	R&D Systems	DY2846
Syndecan-1	R&D Systems	DY2780
Aggrecan	R&D Systems	DY1220
BMP-2	R&D Systems	DY355
BMP-4	R&D Systems	DY314
BMP-9	R&D Systems	DY3209
Rank-L	R&D Systems	DY626
OPN	R&D Systems	DY1433

DOI [to be added]

Periostin	R&D Systems	DY3548B
OCN	R&D Systems	DY1419
MMP-1	R&D Systems	DY901
TIMP-1	R&D Systems	DY970
MMP-2	R&D Systems	DY902
TIMP-2	R&D Systems	DY971
MMP-9	R&D Systems	DY911
MPO	R&D Systems	DMYE00B
IL-1β	R&D Systems	DY201
IL-10	R&D Systems	DY217B
CD14	R&D Systems	DY383
eNOS	R&D Systems	DY950
OPG	R&D Systems	DY805

Histology and Immunohistochemistry

**Reagents/Antibodies**	**Vendor or Source**	**Catalog #**	**Working concentration**
Haematoxylin<br>Eosin	Panreac<br>Bio-Optica	255298.1610<br>05-11007	
Alizarin red	Sigma	A5533-25G	2% (w/v)
Congo red	Merck	1.01641.0001	
Alcian blue	Sigma	B8438-500ML	
Sirius red	Sigma	365548	0.1% (w/v)
Movat	Abcam	Ab245884	
Rantes	Santa Cruz	sc-365826	2 µg/ml
CD45	Santa Cruz	sc-25590	0.006 µg/ml
CD68	Santa Cruz	Sc-17832	4 µg/ml
eNOS	BD biosciences	612393	2.5 µg/ml
CML	Abcam	ab27684	$6x10^{-8}$ µg/ml
MDA	Abcam	ab194225	8 µg/ml
Nitrotyrosine	Santa Cruz	sc-32757	0.04 µg/ml
Mmp-1	Sigma	SAB2108563-100UL	$1x10^{-5}$ µg/ml
Mmp-2	Abcam	ab37150	2 µg/ml
Timp-2	Santa Cruz	sc-56490	0.2 µg/ml
MMP-9	Abcam	ab76003	$1.8x10^{-5}$ µg/ml
Periostin	Santa Cruz	sc-67233	2 µg/ml
Osteocalcin	Santa Cruz	sc-74495	2 µg/ml
BAX	Santa Cruz	sc-493	1 µg/ml
Caspase 3	Cell Signalling	9662	0.49 µg/ml
p53	Santa Cruz	sc-55476	2 µg/ml
Amyloid-β	Dako	M-0872	1/200
Lumican	Santa Cruz	Sc-166871	2 µg/ml

Primers for qPCR analysis

**Gene**	**Primer**	**Sequence (5´to 3´)**
**CHM-1**	Forward	GGAGGAGATGCTCTGTTTGG

DOI [to be added]

	Reverse	GGAAATAGACGCTGGGAACA
**a-SMA**	Forward	ACTGCCTTGGTGTGTGACAATGG
	Reverse	TGGTGCCAGATCTTTTCCATG
**Vimentin**	Forward	CCTTGAACGCAAAGTGGAAT
	Reverse	TTGGCAGCCACACTTTCATA
**IL-6**	Forward	AGTTCCTGCAGAAAAAGGCAAAG
	Reverse	CATTTGCCGAAGAGCCCTCA
**CCL2**	Forward	TCCCAAAGAAGCTGTGATCTTCA
	Reverse	TTTGCTTGTCCAGGTGGTCC
**Col-1a1**	Forward	GGACACAGAGGTTTCAGTGGT
	Reverse	CACCATCATTTCCACGAGCA
**FN**	Forward	GTTATGGAGGAAGCCGAGGT
	Reverse	CGCTCATAAGTGTCACCCACT
**Decorin**	Forward	CCTGATGACCGCGACTTCGAG
	Reverse	TTTGGCACTTTGTCCAGACCC
**OPN**	Forward	CAAACGCCGACCAAGGAAAA
	Reverse	AGCTGCTTTTCCTCAGAACT
**BMP-2**	Forward	TGCGGTCTCCTAAAGGTCG
	Reverse	GGGGTGGGTCTCTGTTTCAG
**BMP-4**	Forward	AGCTTCCACCACGAAGAACAT
	Reverse	AAGCCCCTTTCCCAATCAGG
**Periostin**	Forward	CACTCTTTGCTCCCACCAAT
	Reverse	ATTTCCTTCCAGCGTCTCAA
**Sox-9**	Forward	GAGGAAGTCGGTGAAGAACG
	Reverse	ATCGAAGGTCTCGATGTTGG
**Runx-2**	Forward	GGTTAATCTCCGCAGGTCACT
	Reverse	CACTGTGCTGAAGAGGCTGTT
**TGF-β**	Forward	TACCTGAACCCGTGTTGCTC
	Reverse	CCGGTAGTGAACCCGTTGAT
**CTGF**	Forward	CTCGCGGCTTACCGACTG
	Reverse	GGCTCTGCTTCTCTAGCCTG

DOI [to be added]

**Lumican**	Forward	TGAGCTGGATCTGTCCTATAA
	Reverse	ATCTTGCAGAAGCTCTTTATG
**MMP-1**	Forward	ACATGAGTCTTTGCCGGAGG
	Reverse	AACAAGGTTGACTTTATTCCAAACA
**TIMP-1**	Forward	GGAATGCACAGTGTTTCCCTG
	Reverse	GGAAGCCCTTTTCAGAGCCT
**MMP-2**	Forward	CGACCACAGCCAACTACGAT
	Reverse	GTCAGGAGAGGCCCCATAGA
**TIMP-2**	Forward	GCTGCGAGTGCAAGATCACG
	Reverse	AGAGCTGGACCAGTCGAAAC
**MMP-9**	Forward	CGGTTTGGAAACGCAGATGG
	Reverse	TGGGTGTAGAGTCTCTCGCT
**HPRT**	Forward	TTGCTTTCCTTGGTCAGGCA
	Reverse	ATCCAACACTTCGTGGGGTC
**β-actin**	Forward	GCCGCCAGCTCACCAT
	Reverse	TCGATGGGGTACTTCAGGGT
**GADPH**	Forward	ACCAGCCCCAGCAAGAGCACAAG
	Reverse	TTCAAGGGGTCTACATGGCAACTGF

DOI [to be added]
